# A clicking confinement strategy to fabricate transition metal single-atom sites for bifunctional oxygen electrocatalysis

**DOI:** 10.1126/sciadv.abn5091

**Published:** 2022-03-16

**Authors:** Chang-Xin Zhao, Jia-Ning Liu, Juan Wang, Changda Wang, Xin Guo, Xi-Yao Li, Xiao Chen, Li Song, Bo-Quan Li, Qiang Zhang

**Affiliations:** 1 Beijing Key Laboratory of Green Chemical Reaction Engineering and Technology, Department of Chemical Engineering, Tsinghua University, Beijing 100084, China.; 2 Advanced Research Institute of Multidisciplinary Science, Beijing Institute of Technology, Beijing 100081, China.; 3 School of Materials Science and Engineering, Beijing Institute of Technology, Beijing 100081, China.; 4 National Synchrotron Radiation Laboratory, CAS Center for Excellence in Nanoscience, University of Science and Technology of China, Hefei 230029, Anhui, China.

## Abstract

Rechargeable zinc-air batteries call for high-performance bifunctional oxygen electrocatalysts. Transition metal single-atom catalysts constitute a promising candidate considering their maximum atom efficiency and high intrinsic activity. However, the fabrication of atomically dispersed transition metal sites is highly challenging, creating a need for for new design strategies and synthesis methods. Here, a clicking confinement strategy is proposed to efficiently predisperse transitional metal atoms in a precursor directed by click chemistry and ensure successful construction of abundant single-atom sites. Concretely, cobalt-coordinated porphyrin units are covalently clicked on the substrate for the confinement of the cobalt atoms and affording a Co-N-C electrocatalyst. The Co-N-C electrocatalyst exhibits impressive bifunctional oxygen electrocatalytic performances with an activity indicator Δ*E* of 0.79 V. This work extends the approach to prepare transition metal single-atom sites for efficient bifunctional oxygen electrocatalysis and inspires the methodology on precise synthesis of catalytic materials.

## Teaser

A clicking confinement strategy is proposed to fabricate single-atom catalysts.

## INTRODUCTION

The development of the modern society is propelled by the renewal and iteration of high-efficiency energy storage techniques (*1*–*5*). Beyond the current lithium-ion batteries, rechargeable zinc-air batteries (ZABs) are regarded as promising next-generation energy storage devices because of their high theoretical energy density (1086 Wh kg^−1^, including oxygen) (*6*, *7*), low cost (*8*), inherent safety (using aqueous electrolyte) (*9*, *10*), and potential feasibility to achieve high rates and long cycling life span (*11*). Nevertheless, the cathodic processes of ZABs, referring to oxygen reduction reaction (ORR) during discharge and oxygen evolution reaction (OER) during charge, are highly sluggish in kinetics, rendering large polarization and consequent poor energy efficiency under working conditions (*12*, *13*). Therefore, high-performance bifunctional ORR/OER electrocatalysts are urgently demanded to accelerate the redox kinetics and promote the practical performances for ZABs (*14*).

Noble metal–based electrocatalysts exhibit acceptable electrocatalytic performances, such as Pt-based electrocatalysts for ORR and Ir-based electrocatalysts for OER. However, their high cost and scarcity severely restrict large-scale applications. To address the above issues, tremendous efforts have been devoted to develop noble metal–free electrocatalysts as substitutions, including transition metal compounds (*15*–*18*), heteroatom–doped carbon (*19*–*21*), and their composites (*22*, *23*). Recently, M-N-C (M refers to a transition metal atom) single-atom catalysts (SACs) emerge as promising bifunctional ORR/OER electrocatalysts (*24*, *25*). Typically, transition metal atoms are dispersed at the atomic scale and coordinated with coplanar nonmetal atoms (N or C atoms in most cases) to form well-defined single-atom sites that are further anchored within the carbon substrate (*26*–*28*). M-N-C SACs exhibit extraordinary bifunctional electrocatalytic ORR/OER performances originated from their unique chemical structure. Specifically, the atomic dispersion of the transition metal atoms renders maximum atom efficiency up to 100% (*29*, *30*), the unoccupied *d* orbitals of the center transition metal atoms efficiently interact with oxygen intermediates to ensure ultrahigh intrinsic activity (*31*), and the conductive and porous carbon skeleton contributes to favorable electronic conduction and ion transportation capability (*32*). Therefore, M-N-C SACs serve as the frontier of bifunctional ORR/OER electrocatalysts from the aspect of both fundamental investigation and practical application.

Dispersion of the transition metal atoms at the atomic scale constitutes the key character of the M-N-C SACs that promises high electrocatalytic activity, yet is also the main challenge for their efficient fabrication. M-N-C SACs are usually fabricated via the pyrolysis of precursors containing transition metal, nitrogen, and carbon sources (*33*–*35*). However, the transition metal atoms are easy to aggregate under high temperature and form elemental metal or conventional compounds instead of the desired single-atom sites (*36*, *37*). The main reason is the lack of confinement of the transition metal atoms in the routine precursors, which fail to predisperse the transition metal atoms or to prevent the aggregation of the metal atoms under high temperatures. For instance, simply annealing the precursor consisting of graphene- and cobalt-coordinated porphyrin without efficient confinement on the cobalt atoms led to the formation of metallic cobalt particles instead of desired cobalt single-atom sites (*38*). Therefore, rational precursor design, especially the confinement of transition metal atoms, is regarded as the core for the fabrication of M-N-C SACs.

**Fig. 1.**
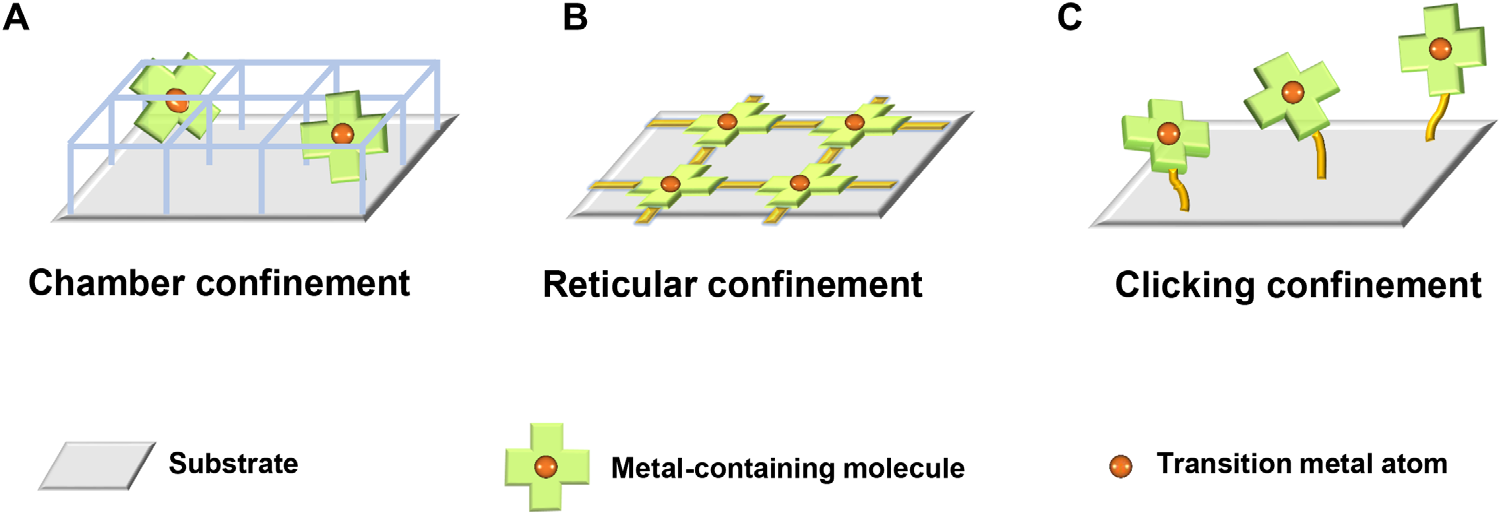
Different confinement strategies to fabricate M-N-C SACs. Schematic representation of the precursor design strategies to fabricate M-N-C SACs regarding (**A**) the chamber confinement strategy, (**B**) the reticular confinement strategy, and (**C**) the clicking confinement strategy.

The currently reported approaches to confine transition metal atoms in the precursor toward the fabrication of M-N-C SACs can be mainly classified into two categories in terms of the chamber confinement strategy and the reticular confinement strategy. Chamber confinement, directed by the principles of supramolecular chemistry, refers to constructing stable local chemical environments to isolate the transition metal precursors. It is usually realized via spatially confining transition metal–containing small molecules separately into the rigid and isolated chambers (Fig. 1A), such as encapsulating metal acetylacetonate into the chamber of zeolite imidazolate frameworks (*29*, *39*–*41*). Chamber confinement puts forward strict requirements on the size relationship between the chamber and the transition metal-containing molecule to ensure full accommodation and effective confinement of a single transition metal atom in a single chamber. Reticular confinement, on the other hand, uses strong coordinate or covalent bonds to stabilize the transition metal atoms under the guidance of reticular chemistry. Typically, reticular confinement can be achieved via weaving symmetrical transition metal–containing small molecules into periodic frameworks with infinite permutation (such as covalently linking metal-porphyrin or metal-phthalocyanine into covalent organic frameworks), where the transition metal atoms are consequently confined at the node of the reticula to be predispersed at the atomic scale (Fig. 1B) (*38*, *42*, *43*). Obviously, reticular confinement makes stringent requirements for the symmetry of the metal-containing small molecules for their periodic tessellation. Despite the above attempts in precursor design, novel confinement strategy is highly considered to break the harsh restrictions on the molecular size or the precursor symmetry required by the above two strategies. New synthesis methodology is expected to enrich the structural design on precursors to afford new opportunities on advanced SAC fabrication.

Here, a clicking confinement strategy is proposed as a new synthesis methodology to confine transition metal atoms in the precursor toward the fabrication of M-N-C SACs. Concretely, clicking confinement refers to preconstructing a stable coordinate environment and further anchoring the obtained molecules onto substrates via covalent linkages following the principles of click chemistry (Fig. 1C). On the one hand, the inherent directivity and saturability of covalent bonds ensure the monodispersion of the grafted transition metal–containing molecules. On the other hand, the relative strong interaction of covalent bonds compared with van der Waals forces prevents the undesired aggregation tendency of the transition metal atoms during pyrolysis. The proposed clicking confinement strategy is not only free from the strict requirements for molecular size or symmetry to bring benefits of broadened synthesis routes but also is of great specificity endowed by the intrinsic advantage of click chemistry to facilitate controllable and directional catalyst synthesis. For the demonstration of the clicking confinement strategy, cobalt porphyrin units are clicked onto a conductive substrate through an amination reaction to form the precursor toward the fabrication of Co-N-C SACs. The obtained Co-N-C SAC exhibits excellent bifunctional ORR/OER electrocatalytic activity with an indicator Δ*E* of 0.79 V, outperforming most of the reported M-N-C SACs. The Co-N-C SAC further enables ZABs with superb electrochemical performances in terms of a low voltage gap of 0.83 V and stable 200 cycles at a high current density of 25 mA cm^−2^. The proposed clicking confinement strategy extends the approach to preparing transition metal single-atom sites for efficient bifunctional oxygen electrocatalysis and high-performance ZABs.

## RESULTS

### Clicking confinement

**Fig. 2.**
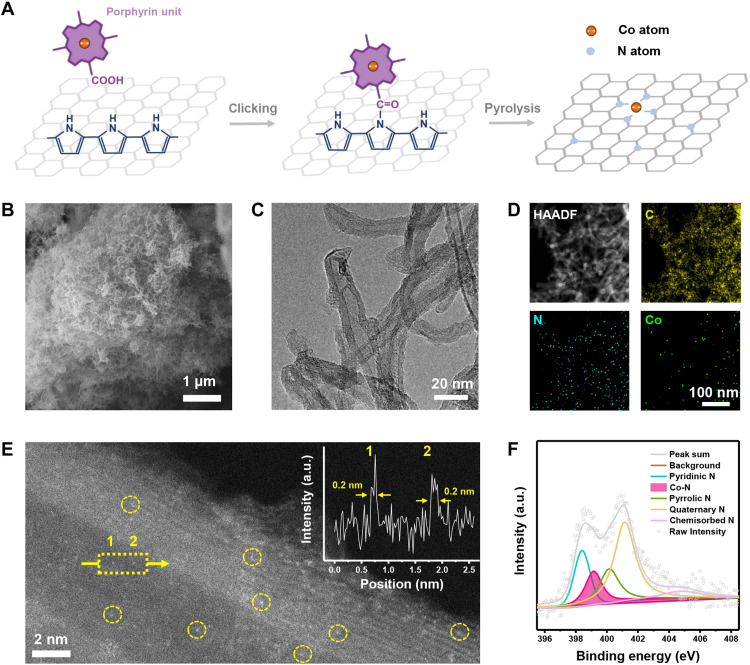
Material characterization of CoNC SAC. (**A**) Schematic of the synthesis procedure of CoNC SAC. (**B**) SEM, (**C**) TEM, and (**D**) HAADF-STEM images and corresponding EDS mapping of CoNC SAC. (**E**) High-resolution HAADF-STEM image of CoNC SAC, where the cobalt single atoms are marked with yellow circles. The inset in (E) is the linear scan analysis along the arrow. (**F**) N 1 s XPS spectrum of CoNC SAC. a.u., arbitrary unit.

Click chemistry, one of the most useful and attractive organic synthesis methodology proposed by Sharpless and co-workers, aims to achieve quick and reliable organic synthesis through the splicing of small units (*44*). The key to click chemistry synthesis is the high-specific and high-selective click reaction to achieve effective splicing between two organic groups. Under the guidance of click chemistry, organic metal–containing molecule can be clicked onto modified substrate to ensure efficient confinement of the metal atoms regarding precursor design to construct single-atom sites, namely, as the proposed clicking confinement strategy. For the demonstration on the clicking confinement strategy, an amidation reaction was selected as the click reaction to graft transition metal–containing molecules onto the conductive substrate considering the high reaction specificity between the carboxyl and the amino groups (*45*, *46*). Cobalt-coordinated porphyrin and carbon nanotubes (CNTs) were selected as the metal-containing molecule and the conductive substrate, respectively (Fig. 2A). It is obvious that the carboxyl and the amino groups should be assigned to the metal-containing molecule and the conductive substrate, respectively, for further amidation clicking. Hence, the carboxyl group was functionalized onto the cobalt-coordinated porphyrin units to afford cobalt(II) meso-tetra(4-carboxyphenyl)porphine (named as CoPor-carboxy) as the reactant for further grafting. Meanwhile, the amino groups are introduced on CNTs via polypyrrole modification considering that polypyrrole has secondary amino (*47*), and the sample was named as CNT-amino. The fabrication of CNT-amino was confirmed by its rough surface compared with the smooth surface of bare CNT (figs. S1 and S2), detectable N element (figs. S3 and S4), and x-ray diffraction (XRD) results (fig. S5).

Armed with the carboxy groups in the CoPor-carboxy units and amino groups in CNT-amino, respectively, the amidation reaction for clicking was subsequently performed to graft cobalt porphyrin onto the substrate with amide bond and afford the pyrolysis precursor (named as CNT-amido-CoPor). CNT-amido-CoPor demonstrated similar morphology (fig. S6) and XRD results (fig. S7) as those of CNT-amino, suggesting that the introduced cobalt porphyrin units are well dispersed without undesired aggregation. According to the deconvoluted N 1s x-ray photoelectron spectroscopy (XPS) spectra, pyrrolic N (originated from the un-amidated pyrrole monomer in polypyrrole), amide N (originated from the amido bond as the result of clicking), and Co-N interaction (originated from the cobalt porphyrin) can be respectively identified to confirm the predesigned structure with cobalt porphyrin grafted onto CNT-amino with amido bonds (fig. S8). The construction of amido bonds can be further verified by the Fourier-transformed infrared spectrometry (FTIR) results, where the peak located at 1650 cm^−1^ can be assigned to the amide I band (υ C═O) of the tertiary amido group in the CNT-amido-CoPor precursor (fig. S9). Moreover, isolated cobalt metal atoms can be easily observed in the high-angle annular dark field scanning transmission electron microscope (HAADF-STEM) images (fig. S10) and corresponding energy-dispersive spectroscopy (EDS) elemental mapping (fig. S11). These results unambiguously indicate that the clicking confinement strategy successfully disperse the cobalt atoms at the atomic scale in the precursor, which is key to the fabrication of M-N-C SACs.

The well-designed precursor with surface clicking was further annealed at 950°C to obtain the final product, which is named as CoNC SAC. Comprehensive material characterizations were performed on CoNC SAC, especially to examine the atomic dispersion of the Co atoms and to reveal the effectiveness of the clicking confinement strategy. Scanning electron microscopy (SEM) and transmission electron microscopy (TEM) images of CoNC SAC indicate a network morphology woven from tubular structure that inherits the morphology characters of CNTs (Fig. 2, B and C, and fig. S12). Such a morphology ensures the axial electron conduction and aperture-induced ion transport capability to render the surface active sites accessible to the electron and ion dual pathways for efficient electrocatalysis. No aggregated cobalt component can be observed in the microscope images, and no peaks corresponding to metallic cobalt or any other cobalt compound can be identified in the XRD patterns or the Raman spectrum of CoNC SAC (figs. S13 and S14). EDS elemental mapping analysis under the STEM mode was additionally carried out. The uniform distribution of carbon, nitrogen, and cobalt elements can be observed (Fig. 2D), further denying the undesired aggregation of the cobalt elements into nanoparticles. The cobalt content was quantitatively measured via multiple characterizations, including EDS analysis (fig. S15), XPS technique (fig. S16), and inductively coupled plasma optical emission spectrometer (ICP-OES) measurement (table S1), to afford the cobalt content of 1.98 weight percent (wt %), 0.41 atom percent (at %), and 1.71 wt %, respectively. The detectable cobalt elements, together with the aggregation-free existence form of the cobalt element, suggest that the cobalt atoms are dispersed at atomic scale to form single-atom sites.

HAADF-STEM is a powerful technique to straightly observe transition metal single atoms in carbon-based materials as the bright dots originated from the Z-contrast differences (*48*, *49*). As is exhibited in Fig. 2E and fig. S17, isolated bright dots are distributed separately on the CNT, indicating that the Co atoms are dispersed within the carbon skeleton to form the desired single-atom sites. Linear scan analysis along the arrow pointed out the full width at half-maximum of the single bright dots being ca. 0.2 nm, matching the diameter of single cobalt atoms. The existence of the Co-N-C sites is additionally verified by the high-resolution nitrogen 1s XPS characterization (Fig. 2F) and nitrogen K-edge x-ray absorption near edge structure (XANES) spectra (fig. S18). Co-N interaction can be clearly identified to indicate that the single cobalt atoms are coordinated with N atoms to form typical Co-N-C active sites. In addition, the cobalt element demonstrates an oxidation state slightly lower than +2, detected by the cobalt 2p XPS spectra (fig. S19) and the cobalt L-edge XANES spectra (fig. S20), which is consistent to the structure of typical Co-N-C single-atom sites (*50*, *51*). The above results unambiguously qualify CoNC SAC as a SAC with atomically dispersed Co-N-C sites and consequently confirm the clicking confinement strategy as a highly effective synthesis methodology to fabricate M-N-C SACs.

### Electrocatalytic performance

**Fig. 3.**
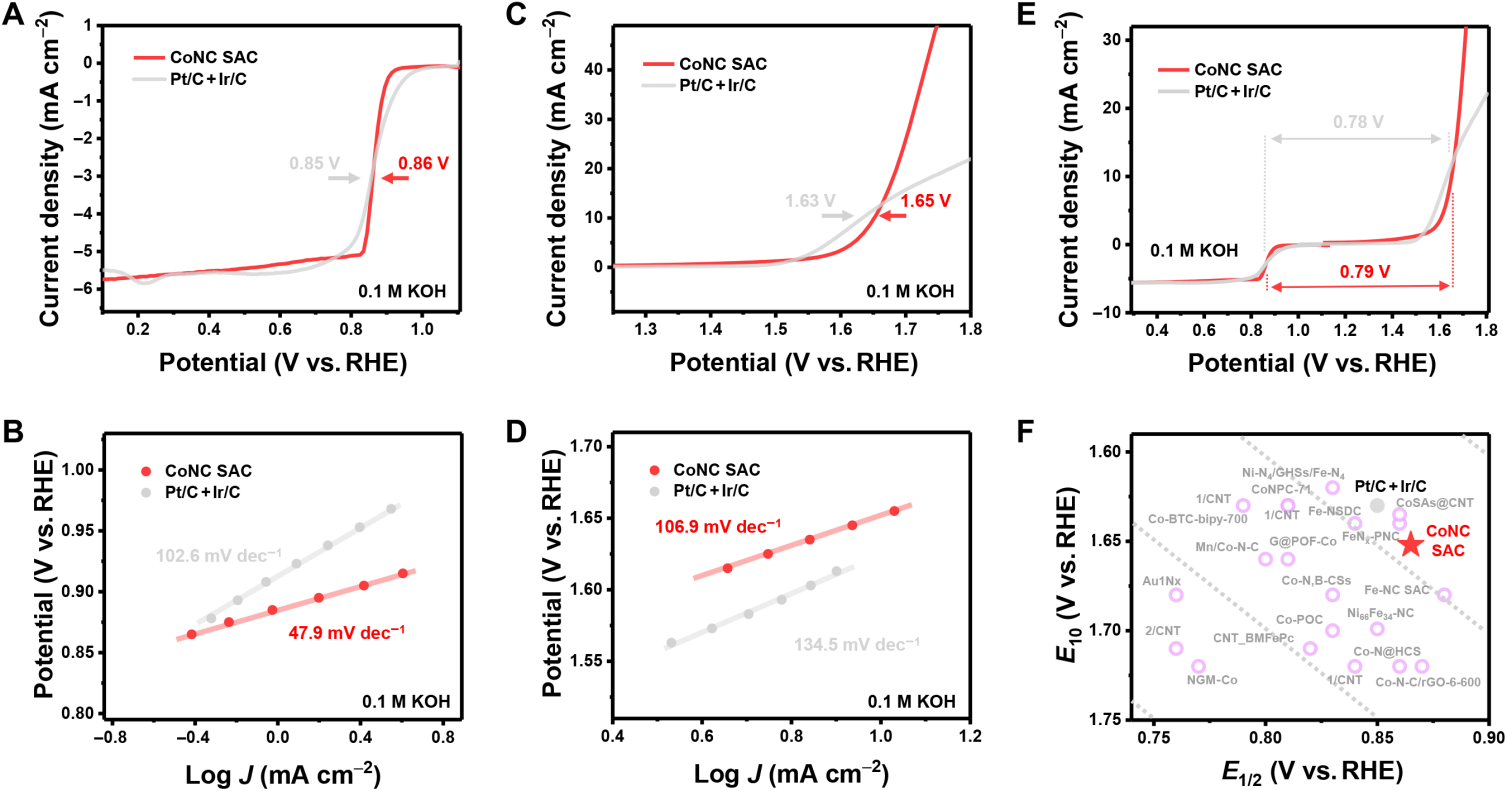
Bifunctional performance evaluation. (**A**) ORR and (**C**) OER LSV profiles of the CoNC SAC and Pt/C + It/C electrocatalysts. (**B**) and (**D**) are the corresponding Tafel plots for ORR and OER, respectively. (**E**) Bifunctional LSV curves of the CoNC SAC and Pt/C + It/C electrocatalysts. (**F**) Diagram for comparing the bifunctional performance of the CoNC SAC, Pt/C + It/C, and other reported electrocatalysts based on M-N-C SACs.

Rechargeable zinc-air batteries starve for the cathodic electrocatalysts to have excellent ORR and OER activity simultaneously. The electrocatalytic performance of the CoNC SAC electrocatalyst for ORR and OER was evaluated under a three-electrode system with oxygen-saturated 0.1 M KOH electrolyte, where the noble metal–based Pt/C + Ir/C electrocatalyst served as the benchmark. All the potentials were calibrated to the reversible hydrogen electrode (RHE). The electrocatalytic ORR activity was evaluated on the basis of the 95% *iR* (*i* refers to current, and *R* refers to resistance)–compensated linear sweep voltammetry (LSV) profiles (Fig. 3A). The onset potential of the CoNC SAC electrocatalyst is 0.93 V versus RHE. The CoNC SAC electrocatalyst also exhibits a half-wave potential (*E*_1/2_) of 0.86 V versus RHE, outperforming the noble metal–based Pt/C + Ir/C electrocatalyst with the *E*_1/2_ of 0.85 V versus RHE. The Tafel plots were additionally calculated on the basis of the LSV data to evaluate the kinetics for ORR electrocatalysis. As is demonstrated in Fig. 3B, low Tafel slope of 47.9 mV dec^−1^ was achieved on the CoNC SAC electrocatalyst, lower than that of the Pt/C + Ir/C electrocatalyst (102.6 mV dec^−1^). Such a result manifests the fast ORR kinetics on the CoNC SAC electrocatalyst. The CoNC SAC electrocatalyst also exhibits a stable electron transfer number (*n*) of ca. 3.7 within the ORR potential range, corresponding to a low H_2_O_2_ selectivity of ca. 10% (fig. S21). Such a result implies the dominant four-electron ORR pathway on the CoNC SAC electrocatalyst. The OER performance was subsequently evaluated. According to the 95% *iR*-compensated OER LSV profiles (Fig. 3C), the CoNC SAC electrocatalyst demonstrated a low potential required to reach current density of 10 mA cm^−2^ (*E*_10_) of 1.65 V versus RHE, which is close to the Pt/C + Ir/C electrocatalyst of 1.63 V versus RHE. The CoNC SAC electrocatalyst also affords low Tafel slope of 106.9 mV dec^−1^ for OER (Fig. 3D), indicating faster OER kinetics compared with that of the Pt/C + Ir/C electrocatalyst (134.5 mV dec^−1^).

Bifunctional ORR/OER performance can be quantified with the Δ*E* index, which is defined as the potential gap between *E*_10_ and *E*_1/2_ and is widely accepted as the electrocatalytic activity indicator (*14*, *52*). Figure 3E presents the LSV curves for bifunctional ORR/OER electrocatalysis for the CoNC SAC and Pt/C + Ir/C electrocatalysts. Quantitatively, the CoNC SAC electrocatalyst exhibits a low Δ*E* of only 0.79 V, demonstrating comparable bifunctional performance with the state-of-the-art noble metal–based Pt/C + Ir/C electrocatalyst (Δ*E* = 0.78 V). Therefore, CoNC SAC is highly considered as a promising substitution for noble metal–based electrocatalyst. The bifunctional performance of the CoNC SAC electrocatalyst is further compared with several reported bifunctional electrocatalysts based on M-N-C SACs (table S2). *E*_1/2_ and *E*_10_ are selected as the horizontal and vertical axis, respectively, in the comparison chart of Fig. 3F. Obviously, the electrocatalyst whose corresponding point is located at the upper right of the figure demonstrates lower Δ*E*, that is, better bifunctional ORR/OER electrocatalytic performance (*53*). Apparently, the CoNC SAC electrocatalyst outperforms most of the reported M-N-C SACs whose Δ*E* is generally higher than 0.80 V. In a word, the obtained CoNC SAC electrocatalyst serves as a promising noble metal–free electrocatalyst to realize excellent bifunctional ORR/OER electrocatalytic performances among the reported M-N-C SACs. The remarkable electrocatalytic performance further manifests the effectiveness of the clicking confinement strategy to fabricate advanced SACs.

### Mechanisms

**Fig. 4.**
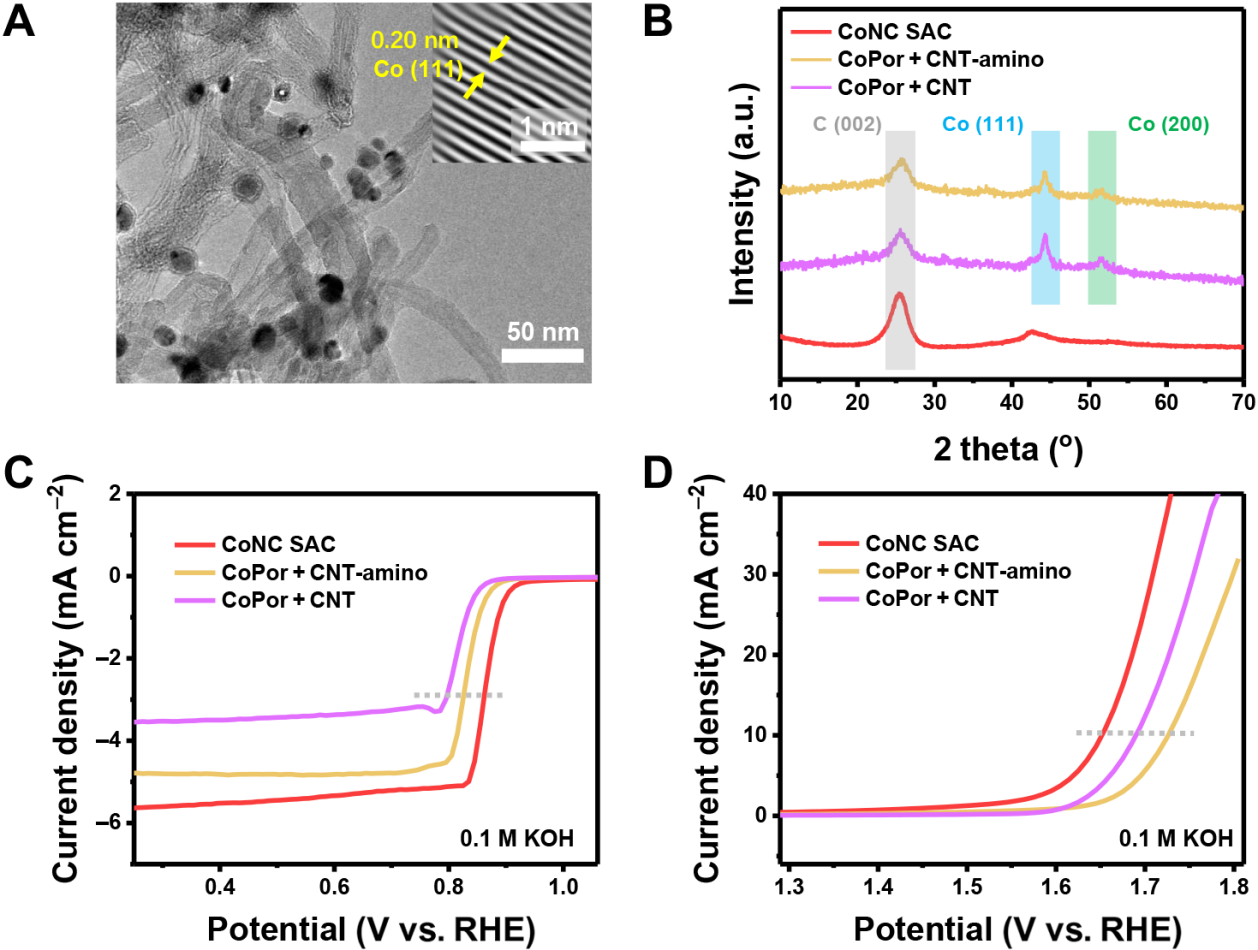
Mechanism investigation for the clicking confining strategy. (**A**) TEM image of CoPor + CNT-amino. The insert is the Fourier-transformed crystalline lattice. (**B**) XRD patterns of CoNC SAC, CoPor + CNT-amino, and CoPor + CNT. (**C**) ORR and (**D**) OER LSV curves of the CoNC SAC, CoPor + CNT-amino, and CoPor + CNT electrocatalysts.

To demonstrate the necessity and effectiveness of the clicking confinement strategy, two control samples without surface clicking were fabricated and evaluated. The first control sample (named as CoPor + CNT-amino) was synthesized via pyrolyzing the precursor consisting of the physical mixture of CNT-amino and cobalt(II) meso-tetraphenylporphine without the covalent clicking linkage between them. The second control sample (named as CoPor + CNT) was fabricated via pyrolyzing the precursor consisting of the physical mixture of CNT and cobalt(II) meso-tetraphenylporphine without the covalent clicking linkage as well. The cobalt contents for the two control samples were well controlled to be around 2.0 wt %, approximately the same as that of CoNC SAC (figs. S22 and S23 and table S1). Without the covalent bonds to confine the cobalt porphyrin units in the precursor, aggregated nanoparticles with a diameter of ca. 10 nm can be directly observed in the TEM images for both of the control samples (CoPor + CNT-amino: Fig. 4A and fig. S24; CoPor + CNT: fig. S25). The XRD results indicate undesired metallic cobalt was formed in CoPor + CNT-amino and CoPor + CNT (Fig. 4B). Furthermore, a lattice fringe with a *d*-spacing of 2.0 Å is observed from the Fourier-transformed crystalline lattice, matching the (111) plane of metallic cobalt. That is, the absence of clicking confinement in the precursor results in undesired aggregation of the cobalt atoms into metallic cobalt nanoparticles instead of atomically dispersed single-atom sites.

Bifunctional ORR/OER performances for the control samples were additionally tested. The CoPor + CNT-amino electrocatalyst exhibits *E*_1/2_ and *E*_10_ of 0.82 and 1.73 V versus RHE, respectively. For the CoPor + CNT electrocatalyst, the *E*_1/2_ and *E*_10_ are 0.79 and 1.69 V versus RHE, respectively (Fig. 4, C and D). The CoNC SAC electrocatalyst outperforms the control samples in both ORR and OER activities, as well as the consequent bifunctional performance (fig. S26). Furthermore, the CoNC SAC electrocatalyst demonstrates the lowest Tafel slopes and charge transfer resistance compared with the control samples in both ORR and OER processes (figs. S27 and S28). The inferior performances of the control samples are reasonable as the aggregation of the cobalt atoms significantly reduces the metal utilization efficiency of the active sites. Compared with the control samples, the successful dispersion of the transition metal sites and the superb electrocatalytic activity manifest the necessity and effectiveness of the clicking confinement strategy to fabricate high-performance SACs. The surface clicking via covalent linkage not only successfully predisperses the transition metal atoms in the precursor due to the inherent directivity and saturability of the covalent bonds but also prevents the aggregation tendency during pyrolysis considering its relative strong interaction compared with van der Waals forces.

### ZAB performance

**Fig. 5.**
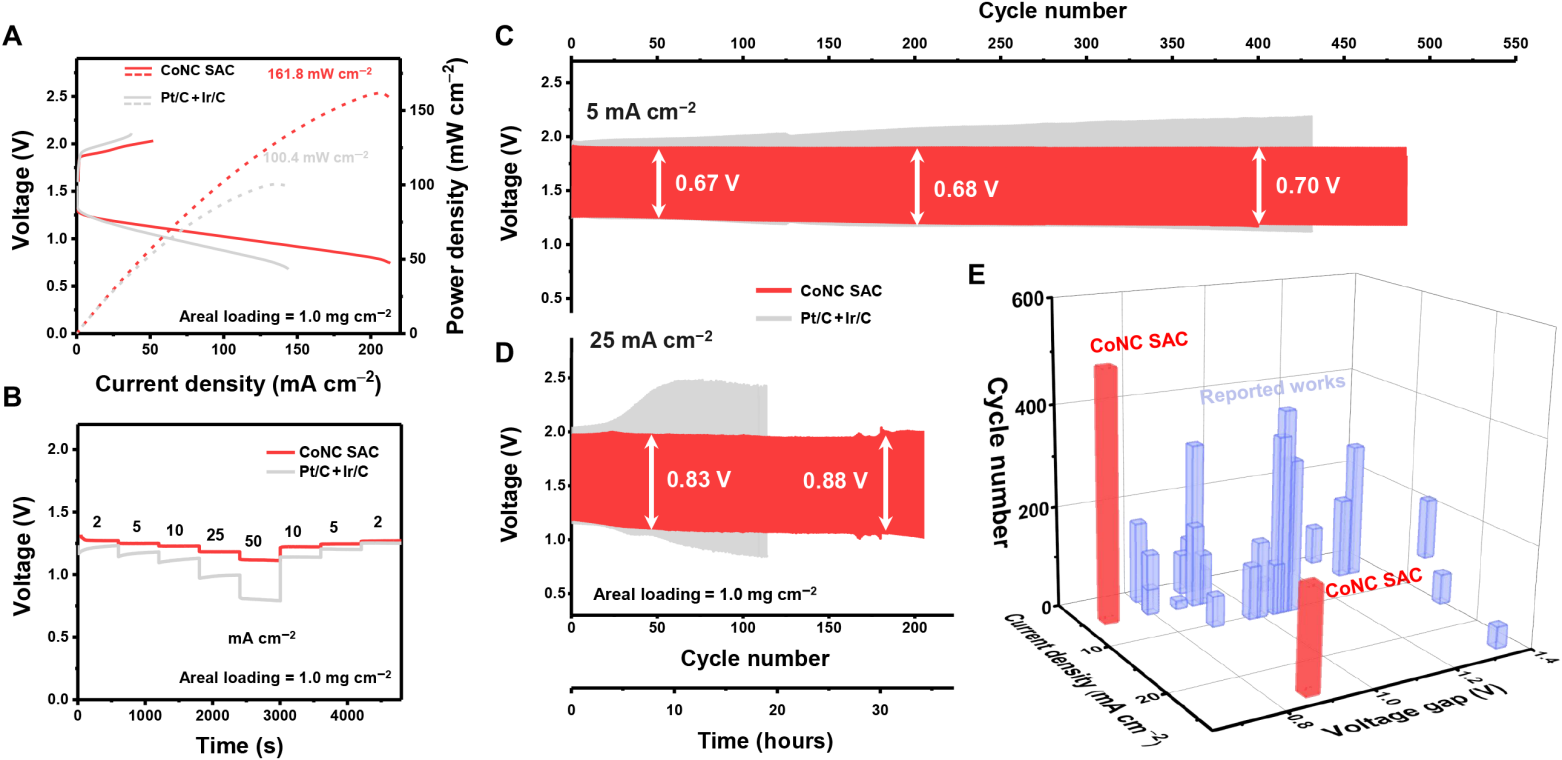
Performance of rechargeable ZABs. (**A**) LSV profiles and discharge power density and (**B**) rate performances of ZABs with the CoNC SAC and Pt/C + It/C electrocatalysts. Galvanostatic cycling curves of ZABs with the CoNC SAC and Pt/C + It/C electrocatalysts at the cycling current density of (**C**) 5.0 and (**D**) 25 mA cm^−2^. (**E**) Rechargeable ZAB performance comparison regarding the cycling current density, voltage gap, and cycle number of the CoNC SAC electrocatalyst and other reported electrocatalysts.

The effective construction of single-atom sites, as well as superb bifunctional ORR/OER performance of the CoNC SAC electrocatalyst, promises its practical application in rechargeable ZABs. Stack-type rechargeable ZABs with the CoNC SAC electrocatalyst loaded on the cathode side were assembled (fig. S29), where ZABs with the noble metal–based Pt/C + Ir/C electrocatalyst were also prepared as a comparison. The areal mass loading of both the electrocatalysts in the cathode was 1.0 mg cm^−2^. According to the polarization profiles (Fig. 5A), the ZABs with the CoNC SAC electrocatalyst exhibit lower polarization for both the charging and the discharging processes compared with the ZABs with the Pt/C + Ir/C electrocatalyst. The CoNC SAC electrocatalyst also enables ZABs with a high peak power density of 161.8 mW cm^−2^, 61.2% higher than that of the Pt/C + Ir/C electrocatalyst. Rate performances were subsequently evaluated via discharging ZABs at different current densities (Fig. 5B). The ZABs with the CoNC SAC electrocatalyst exhibit extraordinary rate performances with higher discharge voltages than the ZAB with Pt/C + Ir/C at all the current densities. Specifically, even at an ultrahigh current density of 50 mA cm^−2^, high discharge voltage of 1.12 V can be achieved. In addition, high discharge voltage of 1.22 V can be recovered when the current density returned to 10 mA cm^−2^, manifesting satisfactory durability of the CoNC SAC electrocatalyst. The specific capacities of the ZABs were additionally evaluated via galvanostatically discharging at 25 mA cm^−2^, where the specific capacities are normalized to the consumed zinc in the anode (fig. S30). The ZABs with the CoNC SAC cathode exhibited a specific capacity of 795 mAh g_Zn_^−1^, close to the theoretical capacity of 821 mAh g_Zn_^−1^.

The long-term durability of the CoNC SAC electrocatalyst was measured via galvanostatic cycling of the ZABs at various current densities. Each discharging-charging cycle was set to be 10.0 minutes. At the cycling current density of 5.0 mA cm^−2^, the ZABs with the CoNC SAC electrocatalyst exhibit long-term working feasibility to afford a 480-cycle life span while maintaining a low voltage gap of 0.70 V (Fig. 5C). As a comparison, the ZABs with the Pt/C + Ir/C electrocatalyst demonstrated unsatisfactory cycling stability with a limited life span. At higher cycling current density of 25 mA cm^−2^, the ZABs with CoNC SAC electrocatalyst also successfully stood for 200 cycles with a low voltage gap of 0.88 V (Fig. 5D), whereas the ZABs with the Pt/C + Ir/C electrocatalyst failed for long-term cycling because of the rapid polarization increasement after the 20th cycle. The electrochemical performances of ZABs with the CoNC SAC electrocatalyst greatly surpass the ZABs with the noble metal–based benchmark and most of the reported noble metal–free electrocatalysts (Fig. 5E and table S3). The impressive performances in working ZABs qualify the CoNC SAC electrocatalyst and further highlight analogous M-N-C SACs as practical electrocatalysts toward the application in the energy-related processes.

## DISCUSSION

To sum up, as a new synthesis methodology to fabricate M-N-C SACs, the proposed clicking confinement strategy is inherently distinct from the conventional chamber confinement strategy and the reticular confinement strategy. The key to the chamber confinement strategy is the supramolecular chemistry that puts forward strict requirements for the compatibility (especially the size relationship) of the host chamber and the guest molecule. For the reticular confinement strategy, reticular chemistry serves as the guiding ideology that the symmetry of metal-containing molecules is strictly restricted for their periodic tessellation into reticula. In comparison, the clicking confinement strategy follows the principles of click chemistry. Three advantages for the clicking confinement strategy are concluded as follows.

1. The clicking confinement strategy breaks the restrictions on molecular size or symmetry and therefore significantly broadens the approaches for M-N-C SAC fabrication. Even the metal-containing molecules with mismatched size or without tri/tetra/hexa-symmetry can function as the metal source to construct M-N-C sites.

2. The clicking confinement strategy only involves surface modifying/grafting at subnanoscale to render excellent capability for morphology retention, which is conducive to morphology design of the catalyst. For instance, tubular morphology for CoNC SAC is reserved from the CNT substrate in this work, whereas, following the chamber confinement strategy or the reticular confinement strategy, undesired morphology evolution is usually accompanied during fabrication.

3. Under the guidance of click chemistry, the clicking confinement strategy is of great specificity originated from the clicking reaction. Hence, the spatial distribution and distribution density of the M-N-C sites, as well as the sequence to introduce different metal atoms, can be well controlled via rationally selecting and matching the functional groups on the transition metal–containing molecules and substrates. The fabrication of Co-N-C SAC in this work serves as a preliminary demonstration, and more M-N-C SACs with other central metal atoms, hetero-coordination sites for coordinating sphere engineering, or even dual-atom sites can be fabricated following the methodology of the clicking confinement strategy.

In conclusion, a clicking confinement strategy is proposed to synthesize M-N-C SACs. Serving as a demonstration, cobalt porphyrin units are covalently clicked onto CNT substrates as the precursor to fabricate Co-N-C SAC. The clicking structure predisperses the Co atoms in the precursor at the atomic scale and prevents undesired aggregation of Co atoms during pyrolysis to endow abundant Co-N-C single-atom sites in the CoNC SAC electrocatalyst. The obtained electrocatalyst exhibits excellent bifunctional ORR/OER performances with Δ*E* = 0.79 V, outperforming most of the analogous bifunctional electrocatalysts. Rechargeable ZABs with the CoNC SAC electrocatalyst afford high-peak power density of 161.8 mW cm^−2^, low charge/discharge polarization, and remarkable stability with long life span of 200 cycles at high current density of 25 mA cm^−2^. The proposed clicking confinement strategy not only expands the methodology to prepare M-N-C SACs but also inspires precise material synthesis with targeted chemical structures toward multiple energy-related applications.

## MATERIALS AND METHODS

### Raw materials

All the raw materials, including pyrrole, ammonium persulfate [(NH_4_)_2_S_2_O_8_; APS], thionyl chloride (SOCl_2_), zinc acetate [Zn(CH_3_COO)_2_], potassium hydroxide (KOH), Pt/C (20 wt % Pt), Ir/C (20 wt % Ir), zinc plate, triethylamine, CoPor-carboxy, cobalt(II) meso-tetraphenylporphine (CoPor), *N*,*N*-dimethylformamide (DMF), *N*-methylpyrrolidone (NMP), 4-dimethylaminopyridine (DMAP), and CNTs (5 wt % in NMP) were purchased from commercial sources and directly used without further purification.

### Synthesis of CNT-amino

Secondary amino group was introduced onto CNT via coaxially surface coating polypyrrole to obtain CNT-amino following an oxidative polymerization reaction. First, 50 ml of NMP and 9.0 g of CNT dispersion were mixed and ultrasonicated for 2.0 hours to form a homogeneous suspension. Meanwhile, 400 μl of pyrrole was dissolved in 100 ml of deionized water. The CNT suspension and the pyrrole solution were mixed and then kept at 4°C for 1.0 hour. Under continuous stirring, 50 ml of 0.06 M APS aqueous solution was added dropwise. Then, the mixture was kept at 4°C under continuous stirring for 24 hours. The product was filtered and then washed with deionized water and ethanol for three times, respectively. CNT-amino was obtained after drying at 80°C for 6.0 hours.

### Synthesis of CNT-amido-CoPor

For the synthesis of CNT-amido-CoPor, cobalt porphyrin units were covalently grafted on CNT-amino after an amidation reaction. Concretely, 126 mg of CoPor-carboxy and 43.2 μl of SOCl_2_ were dissolved in 10 ml of DMF successively and then sonicated for 10 min, during which the carboxyl group was transformed to acid chloride. DMAP (72.9 mg), serving as the catalyst for further amidation, was then added into the above mixture to form the feedstock solution. Meanwhile, 120 mg of CNT-amino was dispersed in 30 ml of DMF followed by the addition of 208 μl of triethylamine and sonication for 45 min to form a homogeneous suspension. Under continuous stirring, the feedstock solution was added into the CNT-amino suspension dropwise. The mixture was kept at 80°C for 24 hours under continuous stirring. After naturally cooling to room temperature, the product was filtered and washed with DMF and ethanol under sonication for three times, respectively. After drying at 60°C for 12 hours, CNT-amido-CoPor was finally obtained. The mass ratio of the grafted Co porphyrin units and CNT-amino was 1:5 calculated on the basis of the mass increase.

### Synthesis of CoNC SAC

CoNC SAC was prepared by the pyrolysis of CNT-amido-CoPor. Concretely, 100 mg of CNT-amido-CoPor was placed in a quartz boat. The quartz boat was then placed in a horizontal quartz tube equipped with a furnace. Under continuous argon flow (200 ml min^−1^), the furnace was heated to 950°C with a heating rate of 5°C min^−1^ and kept at 950°C for 3.0 hours. The furnace was then naturally cooled to room temperature to afford CoNC SAC as the final product.

### Synthesis of CoPor + CNT-amino and CoPor + CNT

CoPor + CNT-amino was synthesized via annealing the mixture of CoPor and CNT-amino. Specifically, 200 mg of CNT-amino was added to 75 ml of ethanol and sonicated for 30 min to afford a homogeneous suspension. Then, 40 mg of CoPor without the carboxy group was dissolved into the above suspension under continuous stirring. The mass ratio of the cobalt porphyrin unites and CNT-amino was the same as that in CNT-amido-CoPor. After sonication for 10 min for thorough mixing, the solvent was evaporated at 60°C for 24.0 hours to afford the intermediate. Under continuous argon flow (200 ml min^−1^), the intermediate was annealed at 950°C for 3.0 hours with a heating rate of 5°C min^−1^ to obtain CoPor + CNT-amino as a control sample. CoPor + CNT was synthesized following otherwise identical procedures as CoPor + CNT-amino except replacing CNT-amino with CNT.

### Material characterization

Morphology characterization was carried out on a JSM 7401F (JEOL Ltd., Tokyo, Japan) SEM operated at 3.0 kV and a JEM 2010 (JEOL Ltd., Tokyo, Japan) TEM operated at 120.0 kV. Aberration-corrected HADDF-STEM images were recorded on an FEI Titan Cubic Themis G2 300 TEM equipped with double spherical aberration correctors and an HADDF detector with the convergence angle being 24 mrad and collection angle between 90 and 240 mrad. The working voltage was 300.0 kV. EDS analysis and corresponding elemental mapping were carried out using the JEM 2010 TEM or the FEI TEM equipped with an Oxford Instrument energy-dispersive x-ray spectrometer. The cobalt element contents were detected by an inductively coupled plasma optical emission spectrometer (IRIS Intrepid II XSP, ThermoFisher, USA). XRD characterization was performed via a Bruker D8 Advanced diffractometer with Cu-K_α_ radiation at 40.0 kV and 120 mA as the x-ray source. FTIR was performed on a NEXUS 870 spectrograph. Raman spectrum was obtained by using a Horiba Jobin Yvon LabRAM HR800 Raman spectrophotometer with a laser excitation wavelength of 532 nm. XPS measurements were carried by using Escalab 250xi. All the samples were cleaned with argon plasma in advance. All the XPS results were calibrated using C 1s line at 284.6 eV. XANES characterizations were carried out from the Beamlines (MCD-A and MCD-B) in Hefei National Synchrotron Radiation Laboratory (NSRL).

### Electrochemical evaluation

Electrocatalytic performances were evaluated using a three-electrode system controlled by a CHI 760E electrochemistry station. The reference electrode and the counter electrode were a saturated calomel electrode (SCE) and a platinum sheet, respectively. The working electrode was a rotating ring-disk electrode (Pine Research Instrument, USA), of which the disk electrode was a glassy carbon electrode with a diameter of 5.0 mm and area of 0.196 cm^2^, and the ring electrode was a platinum ring with an inner diameter of 6.5 mm and an outer diameter of 7.5 mm. All recorded potentials were calibrated to the RHE according to the following equation

$$E_{\text{RHE}}=E_{\text{SCE}}+0.241+0.0592\text{pH}$$ (1)

where *E*_RHE_ and *E*_SCE_ are the potentials relevant to RHE and SCE, respectively.

For the preparation of the working electrode, the electrocatalysts were coated onto the disk electrode following a drop-casting method. Specifically, 10.0 mg of electrocatalyst was mixed with 1.90 ml of ethanol and 100 μl of Nafion solution (5.0 wt % in ethanol), followed by ultrasonication for 10 min to form a homogeneous suspension. Suspension (10.0 μl) was dropped onto the disk electrode, which was mechanically polished and ultrasonically washed by deionized water and ethanol in advance. After full evaporation of the solvent, the working electrode was successfully prepared for electrochemical evaluations. The areal mass loading of the electrocatalyst was 0.25 mg cm^−2^.

All electrochemical evaluations were carried out in O_2_ saturated 0.10 mol liter^−1^ KOH aqueous solution at room temperature. Throughout the measurements, the rotating speed of the working electrode was 1600 rpm. The oxygen electrocatalytic activity was evaluated via 95% *iR*-recompensed LSV measurements performed at a scan rate of 10.0 mV s^−1^ from 0.23 to −1.01 V versus SCE for ORR and from −0.01 to 0.80 V versus SCE for OER. The ring electrode was set at a constant potential of 0.50 V versus SCE during the ORR LSV measurements to detect the hydrogen peroxide as the byproduct.

On the basis of the data collected from the LSV profiles, half-wave potential (*E*_1/2_), defined as the potential to reach half of the limiting current density, served as the index to reveal the ORR activity, while the OER potential at the current density of 10 mA cm^−2^ (*E*_10_) was used to reveal the OER electrocatalytic activity. The potential gap between *E*_1/2_ and *E*_10_ (Δ*E*) was selected to quantify the bifunctional ORR/OER performance. Tafel slopes were calculated on the basis of the LSV profiles according to Tafel equation

$$\eta =\text{blog}(j/j_{0})$$ (2)

where η is the overpotential (η = |*E*_RHE_ – 1.23 V|), *b* is the Tafel slope, *j* is the recorded current density, and *j*_0_ is the exchange current density.

The electron transfer number (*n*) and H_2_O_2_ selectivity were calculated by using the following equations

$$n=4i_{d}/(i_{d}+i_{r}/N)$$ (3)

$$H_{2}O_{2}\text{selectivity}\;(\%)=200\times (i_{r}/N)/(i_{d}+i_{r}/N)$$ (4)

where *i*_d_ and *i*_r_ are the disk and ring currents, respectively, and *N* is the collection efficiency of the ring electrode that was determined to be 0.26.

The electrochemical impedance spectroscopy evaluation was performed at −0.17 or 0.63 V versus SCE for the electrocatalytic ORR and OER processes, respectively. The spectra were recorded with a voltage amplitude of 5.0 mV in a frequency range from 10^−1^ to 10^5^ Hz.

### Assembly of rechargeable ZABs

Rechargeable zinc-air batteries were assembled following a stack-type cell configuration. The anode was a prepolished zinc foil with a diameter of 19.0 mm and thickness of 0.25 mm. The current collectors for both the anode and the cathode were nickel foam (NF). The electrolyte was 6.0 M KOH aqueous solution containing 0.20 M Zn(CH_3_COO)_2_.

The air cathode was a composite of a hydrophilic catalyst layer and a hydrophobic gas diffusion layer (GDL). The hydrophilic catalyst layer was a carbon cloth (CC) substrate (1.5 cm by 1.5 cm, WOS1002, CeTech), and the GDL was a conductive carbon paper (2.0 cm by 2.0 cm, LYS-30T). First, the GDL and the bare CC were mechanically hot pressed at 80°C for 120 s to construct the composite air cathode. After that, the electrocatalysts were loaded on the CC substrate following the same drop casting method for working electrode preparation discussed above. The areal mass loading of all the electrocatalysts was 1.0 mg cm^−2^ for ZAB tests.

### Rechargeable ZAB tests

All the electrochemical evaluations on ZABs were performed under ambient atmosphere at room temperature. The LSV curves were recorded at a scan rate of 10 mV s^−1^ and on the CHI 760E electrochemistry station. The discharge power density (*P*) was calculated according to the LSV profiles using the following equation

$$P=U_{d}\times j_{d}$$ (5)

where *U*_d_ and *j*_d_ are the discharge voltage and discharge current density, respectively.

The rate performance of ZABs was tested by galvanostatically discharging the ZABs at a series of current densities of 2, 5, 10, 25, and 50 and back to 10, 5, and 2 mA cm^−2^, respectively. Galvanostatic discharging-charging evaluation was performed using a Land CT2001 multichannel battery tester. The ZABs were discharged for 5.0 min and then charged for 5.0 min at a given current density of 5 or 10 mA cm^−2^ in each cycle. The specific capacities of ZABs were evaluated on the basis of the galvanostatic discharge results at 25 mA cm^−2^, which were normalized to the mass of consumed zinc.

## Supplementary Materials

### This PDF file includes:

Figs. S1 to S30 Tables S1 to S3 References
