# Supplementary material for: A clicking confinement strategy to fabricate transition metal single-atom sites for bifunctional oxygen electrocatalysis
Source: Sci Adv. 2022 Mar 16;8(11):eabn5091. doi: 10.1126/sciadv.abn5091 (PMC8926326; doi:10.1126/sciadv.abn5091)
Supplement: Supplementary file 1 — Figs. S1 to S30 Tables S1 to S3 References [file sciadv.abn5091_sm.pdf]

Science Advances
AAAS

Supplementary Materials for

A clicking confinement strategy to fabricate transition metal single-atom sites for bifunctional oxygen electrocatalysis

Chang-Xin Zhao, Jia-Ning Liu, Juan Wang, Changda Wang, Xin Guo, Xi-Yao Li, Xiao Chen, Li Song, Bo-Quan Li*, Qiang Zhang*

*Corresponding author. Email: libq@bit.edu.cn (B.-Q.L.); zhang-qiang@mails.tsinghua.edu.cn (Q.Z.)

Published 16 March 2022, *Sci. Adv.* **8**, eabn5091 (2022)
DOI: 10.1126/sciadv.abn5091

This PDF file includes:

Figs. S1 to S30
Tables S1 to S3
References

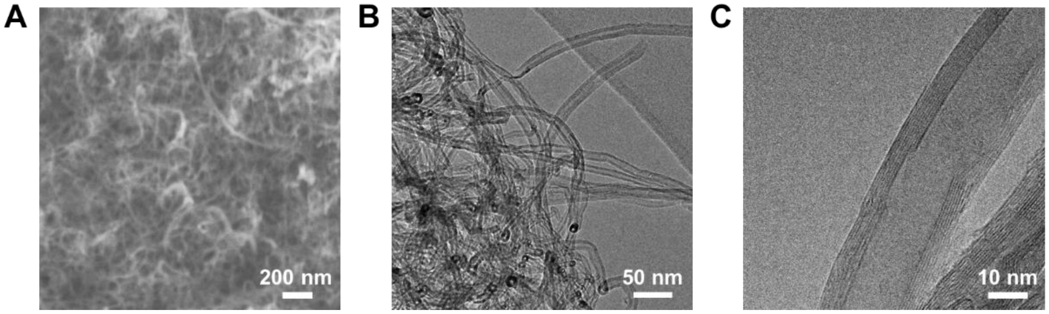

fig. S1. Morphology characterization of CNTs. (A) SEM, (B) and (C) TEM images of CNTs.

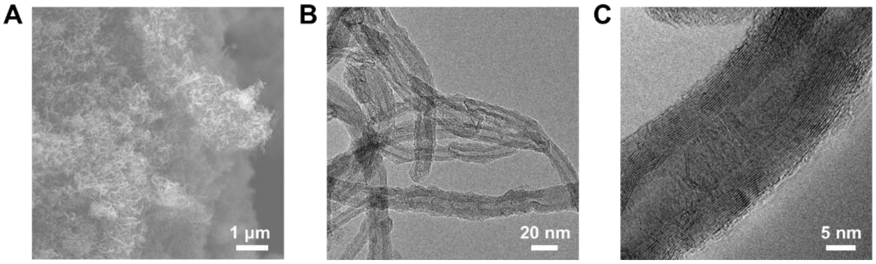

fig. S2. Morphology characterization of CNT-amino. (A) SEM, (B) and (C) TEM images of CNT-amino.

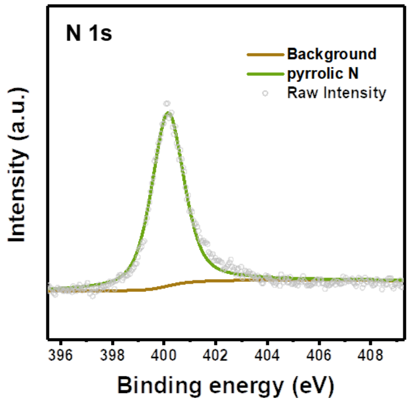

fig. S3. Deconvoluted nitrogen 1s XPS spectrum of CNT-amino. The nitrogen content of CNT-amino is dominant pyrrolic nitrogen, which agrees with the polypyrrole content.

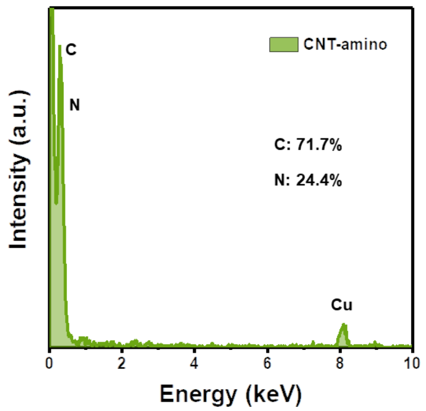

fig. S4. EDS patterns and detected element contents of CNT-amino.

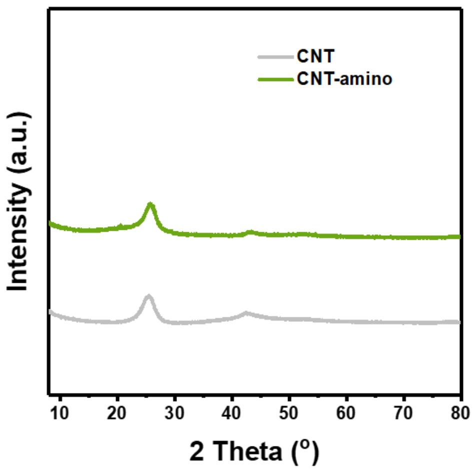

fig. S5. XRD patterns of CNT and CNT-amino.

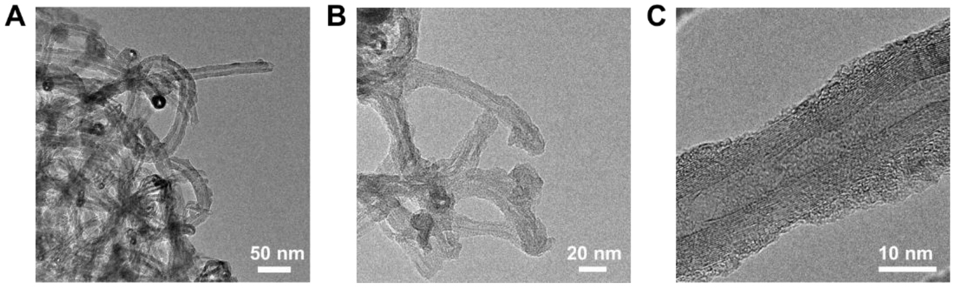

fig. S6. Morphology characterization of CNT-amido-CoPor. (A–C) TEM images of CNT-amido-CoPor.

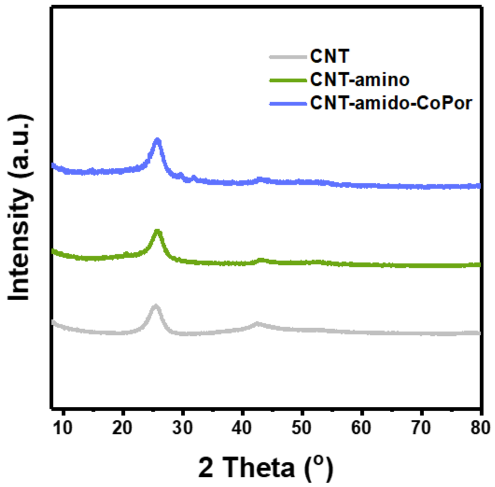

fig. S7. XRD patterns of CNT, CNT-amino, and CNT-amido-CoPor.

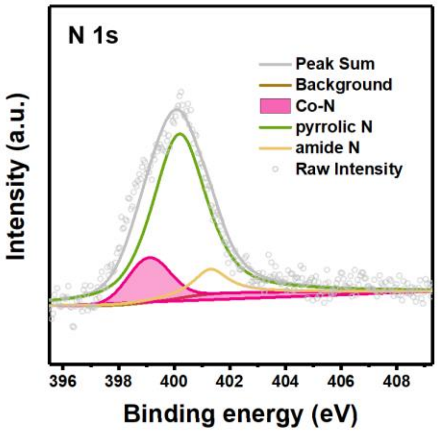

fig. S8. Deconvoluted nitrogen 1s XPS spectrum of CNT-amido-CoPor.

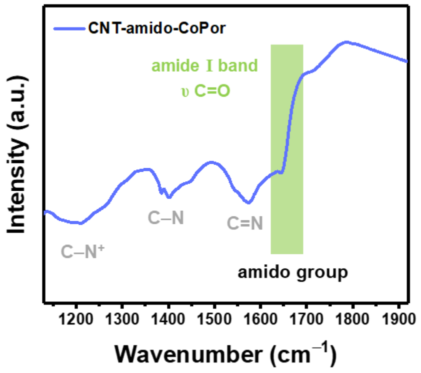

fig. S9. FTIR spectrum of CNT-amido-CoPor. Amido group can be identified in the CNT-amido-CoPor precursor to indicate successful grafting of cobalt porphyrin unit onto the substrate via click reaction.

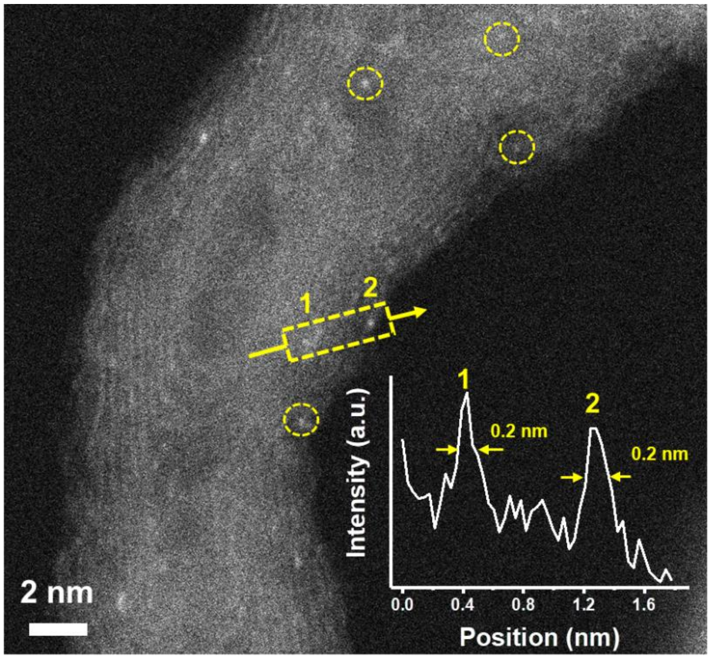

fig. S10. High-resolution HAADF-STEM image of CNT-amido-CoPor. The pre-dispersed Co single-atoms are marked with yellow circles. The inset is the linear scan analysis along the arrow.

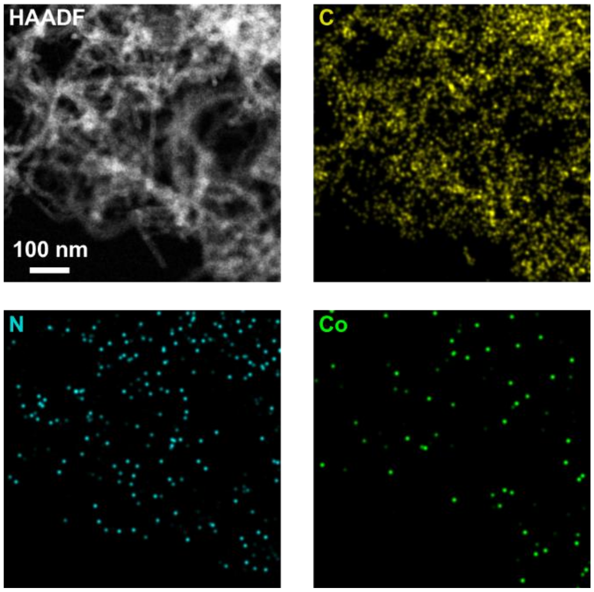

fig. S11. HAADF-STEM and corresponding EDS mapping images of CNT-amido-CoPor.

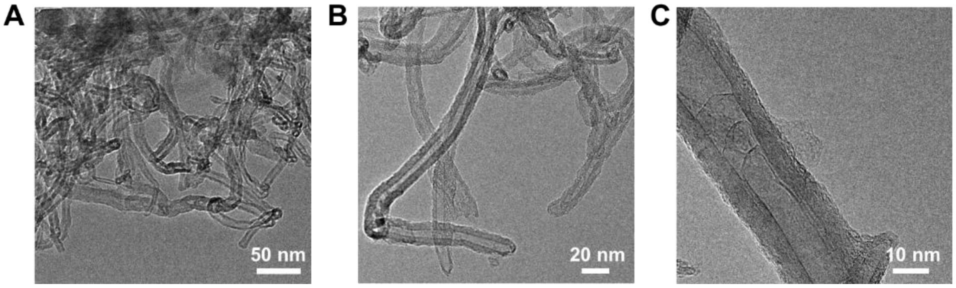

fig. S12. Morphology characterization of CoNC SAC. (A–C) TEM images of CoNC SAC.

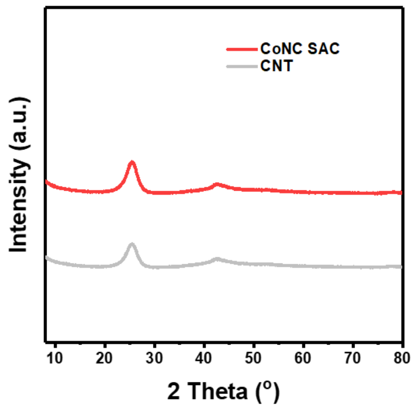

fig. S13. XRD patterns of CNT and CoNC SAC.

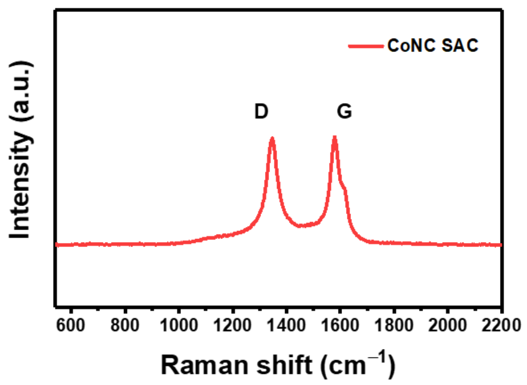

fig. S14. Raman spectrum of CoNC SAC. No peak located at 675 cm^{-1} (corresponding to metallic cobalt) can be identified, denying the existence of metallic cobalt.

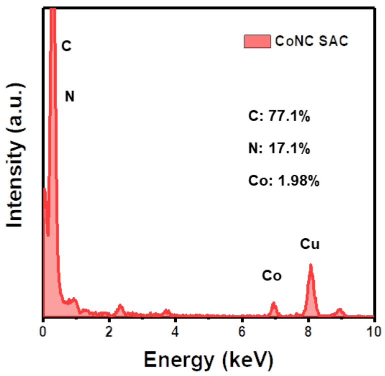

fig. S15. EDS patterns and detected element contents of CoNC SAC.

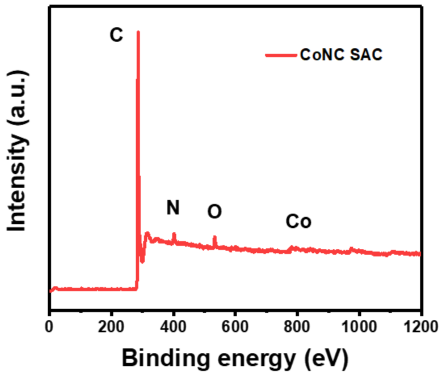

fig S16. XPS survey spectrum of CoNC SAC.

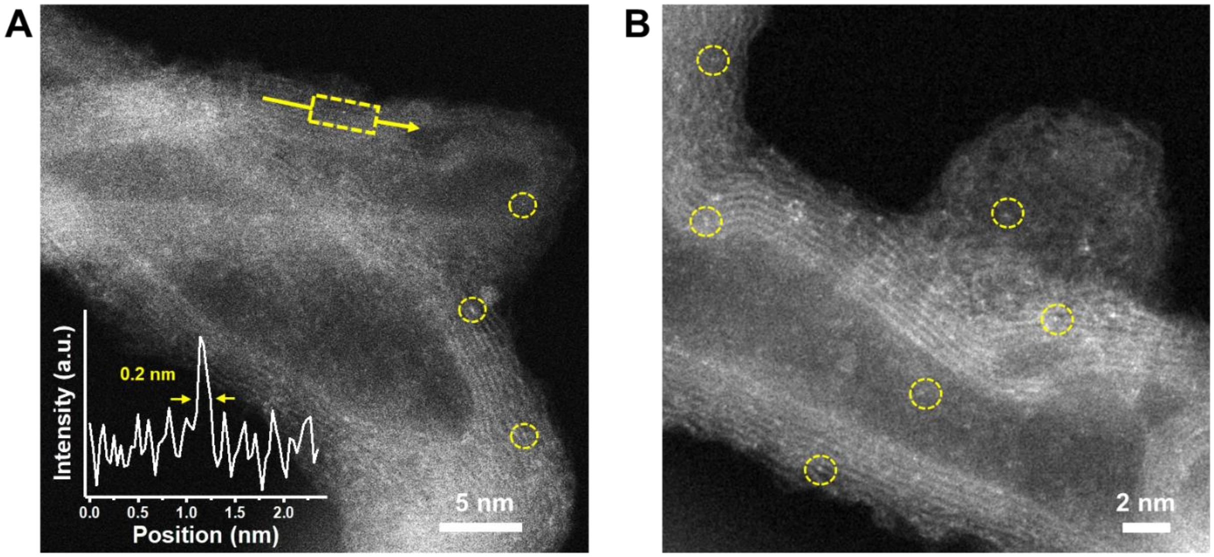

fig. S17. High-resolution HAADF-STEM images of CoNC SAC. The Co single-atoms are marked with yellow circles. The inset in (A) is the linear scan analysis along the arrow.

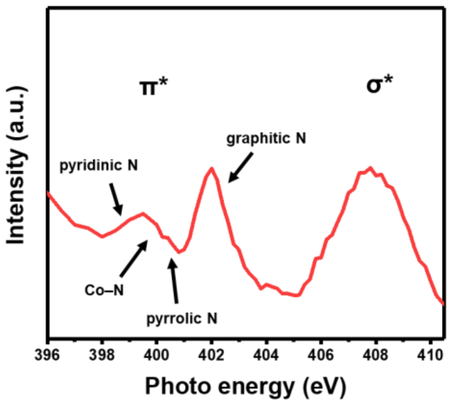

fig. S18. Nitrogen K-edge XANES spectrum of CoNC SAC.

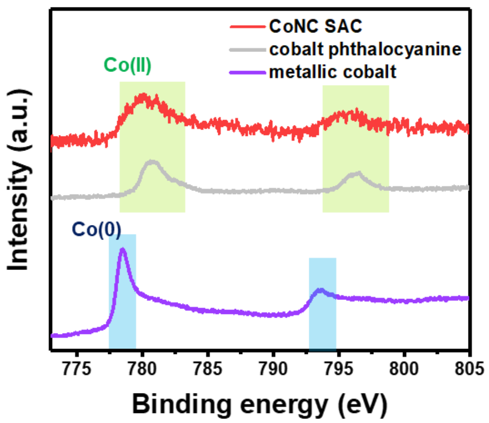

fig. S19. High-resolution cobalt 2p XPS spectra of CoNC SAC, metallic cobalt, and cobalt phthalocyanine.

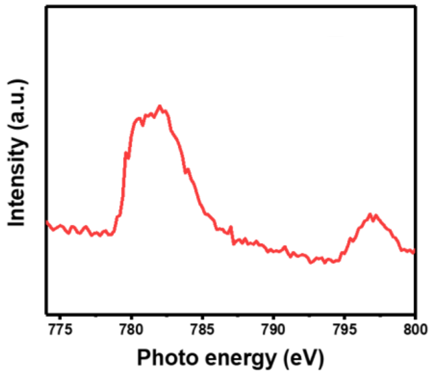

fig. S20. Cobalt L-edge XANES spectrum of CoNC SAC.

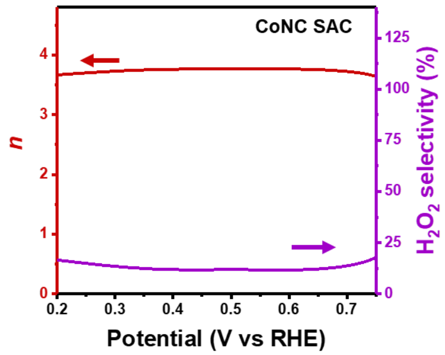

fig. S21. Electron transfer number and H_2O_2 selectivity of the CoNC SAC electrocatalyst.

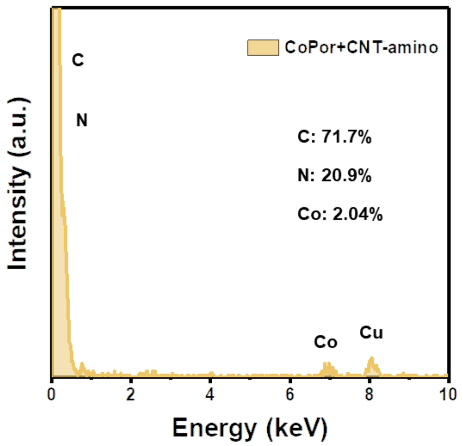

fig. S22. EDS patterns and detected element contents of CoPor+CNT-amino.

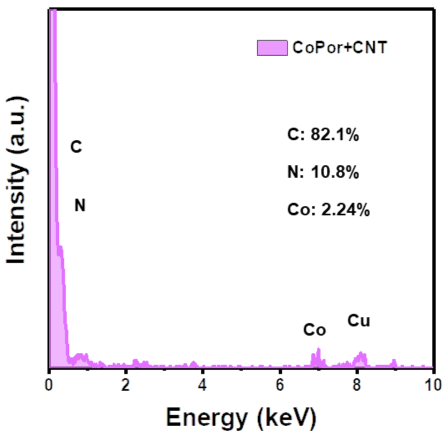

fig. S23. EDS patterns and detected element contents of CoPor+CNT.

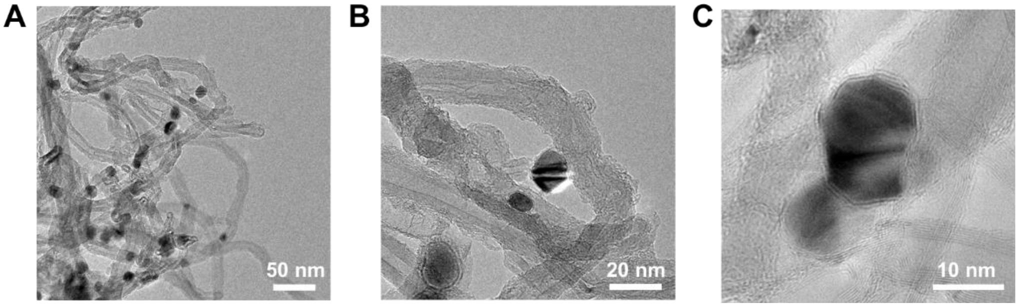

fig. S24. TEM images of CoPor+CNT-amino.

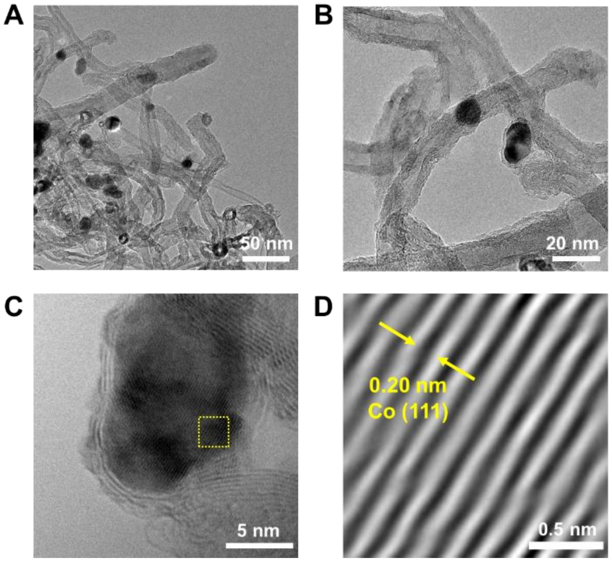

fig. S25. TEM images of CoPor+CNT.

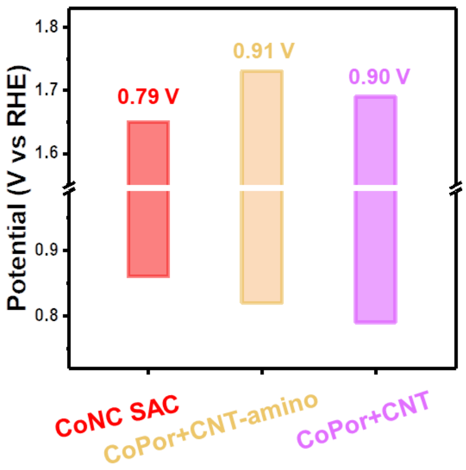

fig. S26. Diagram for comparing the bifunctional electrocatalytic performance of the CoNC SAC, CoPor+CNT-amino, and CoPor+CNT electrocatalysts.

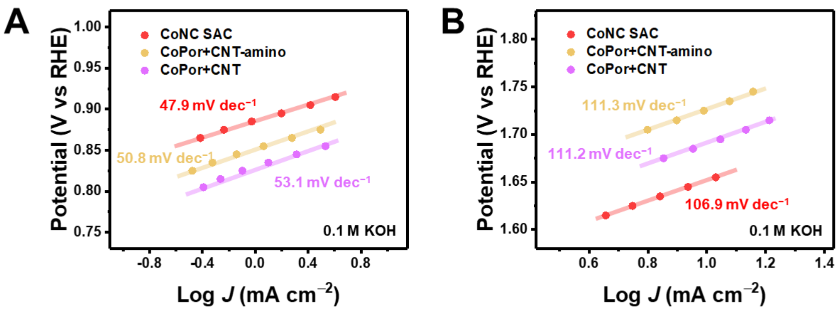

fig. S27. Tafel plots of ORR/OER processes. (A) ORR and (B) OER Tafel plots of the CoNC SAC, CoPor+CNT-amino, and CoPor+CNT electrocatalysts.

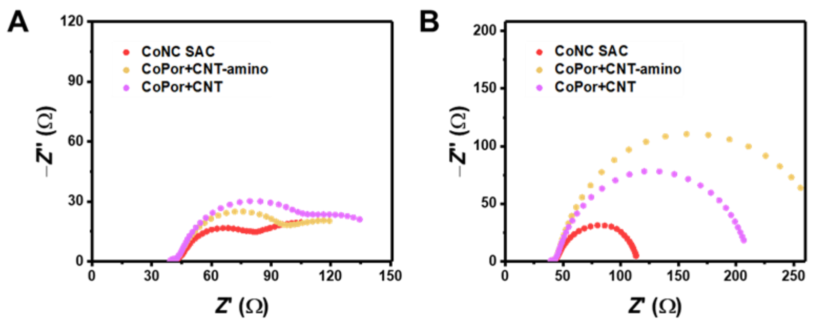

fig. S28. EIS results of ORR/OER processes. EIS spectra of the CoNC SAC, CoPor+CNT-amino, and CoPor+CNT electrocatalysts under (A) ORR and (B) OER conditions.

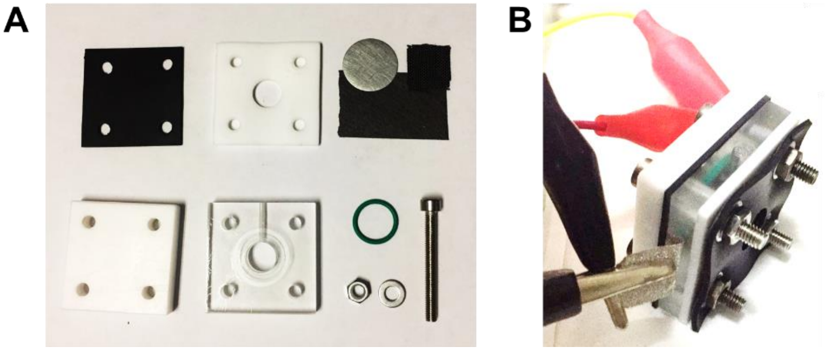

fig. S29. Optical images of zinc–air battery. Optical images of (A) the assembly components of a zinc–air battery and (B) an assembled zinc–air battery.

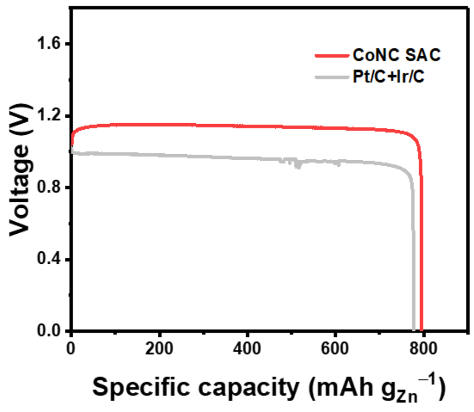

fig. S30. Galvanostatic discharge curves for capacity evaluation. Galvanostatic discharge curves at 25 mA cm^{-2} of ZABs with the CoNC SAC or Pt/C+Ir/C electrocatalysts for capacity evaluation, where the specific capacity was normalized to the mass of consumed zinc anodes.

table S1. Summary of the cobalt content in CoNC SAC, CoPor+CNT-amino, and CoPor+CNT based on ICP–OES and EDS characterization measurements.

Characterization	CoNC SAC	CoPor+CNT-amino	CoPor+CNT
ICP–OES	1.71 wt.%	1.72 wt.%	1.64 wt.%
EDS	1.98 wt.%	2.04 wt.%	2.24 wt.%

table S2. Bifunctional performance comparison of the CoNC SAC electrocatalyst and control samples, noble-metal-based Pt/C+Ir/C, and other reported bifunctional electrocatalysts based on M–N–C single-atom electrocatalytic sites. All the electrocatalytic activity was tested in O_2 saturated 0.10 mol L^{-1} KOH electrolyte.

Electrocatalyst	$E_{1/2}$ (V vs RHE)	E_{10} (V vs RHE)	ΔE (V)	Tafel slope (mV dec^{-1})		Loading (mg cm^{-2})	Reference
				ORR	OER		
**CoNC SAC**	0.86	1.65	0.79	47.9	106.9	0.25	this work
CoPor+CNT-amino	0.82	1.73	0.91	50.8	111.3	0.25	
CoPor+CNT	0.79	1.69	0.90	53.1	111.2	0.25	
Pt/C+Ir/C	0.85	1.63	0.78	102.6	131.2	0.25	
Co-POC	0.83	1.70	0.87	53.5	139	0.1	*(38)*
CNT_BMFePc	0.82	1.71	0.89	/	/	0.11	*(54)*
Co-N@HCS	0.86	1.72	0.86	56	81	0.3	*(55)*
CoSAs@CNT	0.86	1.64	0.78	99	85	0.3	*(56)*
Au_1N_x	0.76	1.68	0.92	74	112	0.375	*(57)*
CoNPC-71	0.81	1.63	0.82	/	/	0.4	*(58)*
Co-BTC-bipy-700	0.79	1.63	0.84	53	77	0.05	*(59)*
Ni-N_4/GHSs/Fe-N_4	0.83	1.62	0.79	55	81	0.25	*(60)*
FeN_x-PNC	0.86	1.635	0.775	/	80	0.14	*(61)*
$Ni_{66}Fe_{34}$-NC	0.85	1.699	0.849	107	120	0.25	*(62)*
Co-N-C/rGO-6-600	0.87	1.72	0.85	54	57	/	*(63)*
Fe-NC SAC	0.88	1.68	0.80	114	65	0.16	*(64)*
Co-N-PDEB	0.84	1.92	1.08	/	153	0.175	*(65)*
NGM-Co	0.77	1.72	0.95	58	/	0.25	*(66)*
Mn/Co-N-C	0.80	1.66	0.86	77	145	0.25	*(67)*
Fe-N_x-C	0.91	1.83	0.92	69	243	0.3	*(68)*
Co-N,B-CSs	0.83	1.66	0.83	64	/	0.1	*(69)*

G@POF-Co	0.81	1.66	0.85	46.9	/	0.1	*(70)*
Fe-NSDC	0.84	1.64	0.80	56	59	ORR: 0.1<br>OER: 0.2	*(71)*
1/CNT	0.81	1.63	0.82	36.9	60.3	0.25	*(72)*
2/CNT	0.76	1.71	0.95	43.5	71.6	0.25	*(72)*
1/CNT	0.84	1.72	0.88	/	84	ORR:<br>0.08<br>OER: 0.2	*(73)*

table S3. Comparison of zinc–air battery performances with CoNC SAC, Pt/C+Ir/C, and other reported electrocatalysts.

Electrocatalyst	Current density (mA cm^{-2})	Voltage gap (V)	Cycle number	Loading (mg cm^{-2})	Reference
CoNC SAC	5	0.70	480	1.0	this work
CoNC SAC	25	0.88	200	1.0	
Pt/C+Ir/C	5	>1.0	430	1.0	
Pt/C+Ir/C	25	>1.5	110	1.0	
PBSCF-NF	10	1.0	150	/	*(74)*
Ag-MnO_2	5	1.3	270	2.6	*(75)*
MnO_2/Co_3O_4	15	1.4	60	2.0	*(76)*
NiO/CoO TINWs	1	0.95	100	0.46	*(77)*
NGM-Co	5	1.09	20	0.5	*(66)*
$Co(OH)_2$+N	1	1.29	75	1.0	*(78)*
Fe/N-C	10	1.04	100	2.2	*(79)*
$S_{5.84\%}$-LCO	2	0.95	300	2.0	*(80)*
CMO/S-300	5	0.8	120	0.8	*(81)*
Co_9S_8/P@CS	5	0.87	1050	1.0	*(82)*
FeCoOOH-NS-3D-FeNC	10	1.06	400	0.25	*(83)*
Ni_3Fe/N-C	10	0.98	105	/	*(84)*
Ni|MnO/CNF	10	1.05	350	1.0	*(85)*
B,N-carbon	5	0.8	50	1.0	*(86)*
NiS_x/NMC-1.5	10	1.08	300	/	*(87)*
Co_9S_8/NSG g-C_3N_4	5	0.8	50	10	*(88)*
CoNMC-700-1	2	0.97	40	/	*(89)*
CCO@C	2	0.81	160	1.0	*(90)*
$Fe_{0.5}Co_{0.5}O_x$/NrGO	10	0.89	60	1.0	*(91)*
NiFe-LDH/Co,NCNF	25	1.35	42	1.0	*(23)*

Co-POC	2	1.2	240	0.1	*(38)*
Co-POC	5	1.3	160	0.1	*(38)*
o-CC-H_2	2	0.92	80	/	*(92)*
Co-N,B-CSs	5	1.55	128	0.2	*(69)*
S,N-Fe/N/ C-CNT	5	0.93	100	1.25	*(93)*
MnSAC	5	0.91	160	1.0	*(25)*

REFERENCES AND NOTES

1. K. Mizushima, P. C. Jones, P. J. Wiseman, J. B. Goodenough, Li_xCoO_2 ($0<x<-1$): A new cathode material for batteries of high energy density. *Mater. Res. Bull.* **15**, 783–789 (1980).

2. J. M. Kim, A. Jo, K. A. Lee, H. J. Han, Y. J. Kim, H. Y. Kim, G. R. Lee, M. Kim, Y. Park, Y. S. Kang, J. Jung, K. H. Chae, E. Lee, H. C. Ham, H. Ju, Y. S. Jung, J. Y. Kim, Conformation-modulated three-dimensional electrocatalysts for high-performance fuel cell electrodes. *Sci. Adv.* **7**, eabk0919 (2021).

3. A. Manthiram, X. Yu, S. Wang, Lithium battery chemistries enabled by solid-state electrolytes. *Nat. Rev. Mater.* **2**, 16103 (2017).

4. D. Kundu, E. Talaie, V. Duffort, L. F. Nazar, The emerging chemistry of sodium ion batteries for electrochemical energy storage. *Angew. Chem. Int. Ed.* **54**, 3431–3448 (2015).

5. W. Cheng, H. Zhang, D. Luan, X. W. Lou, Exposing unsaturated Cu_1–O_2 sites in nanoscale Cu-MOF for efficient electrocatalytic hydrogen evolution. *Sci. Adv.* **7**, eabg2580 (2021).

6. B. J. Hopkins, C. N. Chervin, J. W. Long, D. R. Rolison, J. F. Parker, Projecting the specific energy of rechargeable zinc–air batteries. *ACS Energy Lett.* **5**, 3405–3408 (2020).

7. Y. Li, H. Dai, Recent advances in zinc–air batteries. *Chem. Soc. Rev.* **43**, 5257–5275 (2014).

8. H.-F. Wang, Q. Xu, Materials design for rechargeable metal–air batteries. *Matter* **1**, 565–595 (2019).

9. J. Fu, Z. P. Cano, M. G. Park, A. Yu, M. Fowler, Z. Chen, Electrically rechargeable zinc–air batteries: Progress, challenges, and perspectives. *Adv. Mater.* **29**, 1604685 (2017).

10. X. F. Lu, Y. Fang, D. Luan, X. W. D. Lou, Metal–organic frameworks derived functional materials for electrochemical energy storage and conversion: A mini review. *Nano Lett.* **21**, 1555–1565 (2021).

11. C.-X. Zhao, J.-N. Liu, J. Wang, D. Ren, J. Yu, X. Chen, B.-Q. Li, Q. Zhang, A ΔE=0.63 V bifunctional oxygen electrocatalyst enables high-rate and long-cycling zinc–air batteries. *Adv. Mater.* **33**, 2008606 (2021).

12. W. Xiong, H. Li, H. You, M. Cao, R. Cao, Encapsulating metal organic framework into hollow mesoporous carbon sphere as efficient oxygen bifunctional electrocatalyst. *Natl. Sci. Rev.* **7**, 609–619 (2020).

13. X. F. Lu, B. Y. Xia, S.-Q. Zang, X. W. Lou, Metal–organic frameworks based electrocatalysts for the oxygen reduction reaction. *Angew. Chem. Int. Ed.* **59**, 4634–4650 (2020).

14. C.-X. Zhao, J.-N. Liu, J. Wang, D. Ren, B.-Q. Li, Q. Zhang, Recent advances of noble-metal-free bifunctional oxygen reduction and evolution electrocatalysts. *Chem. Soc. Rev.* **50**, 7745–7778 (2021).

15. G. Li, Q. Xu, W. Shi, C. Fu, L. Jiao, M. E. Kamminga, M. Yu, H. Tueysuez, N. Kumar, V. Suess, R. Saha, A. K. Srivastava, S. Wirth, G. Auffermann, J. Gooth, S. Parkin, Y. Sun, E. Liu, C. Felser, Surface states in bulk single crystal of topological semimetal $Co_3Sn_2S_2$ toward water oxidation. *Sci. Adv.* **5**, eaaw9867 (2019).

16. D. M. Morales, M. A. Kazakova, S. Dieckhoefer, A. G. Selyutin, G. V. Golubtsov, W. Schuhmann, J. Masa, Trimetallic Mn-Fe-Ni oxide nanoparticles supported on multi-walled carbon nanotubes as high-performance bifunctional ORR/OER electrocatalyst in alkaline media. *Adv. Funct. Mater.* **30**, 1905992 (2020).

17. Y. Zhao, L. Xu, L. Mai, C. Han, Q. An, X. Xu, X. Liu, Q. Zhang, Hierarchical mesoporous perovskite $La_{0.5}Sr_{0.5}CoO_{2.91}$ nanowires with ultrahigh capacity for Li–air batteries. *Proc. Natl. Acad. Sci. U.S.A.* **109**, 19569–19574 (2012).

18. X. F. Lu, S. L. Zhang, E. Shangguan, P. Zhang, S. Gao, X. W. Lou, Nitrogen-doped cobalt pyrite yolk–shell hollow spheres for long-life rechargeable Zn–air batteries. *Adv. Sci.* **7**, 2001178 (2020).

19. Q. Wang, Y. Ji, Y. Lei, Y. Wang, Y. Wang, Y. Li, S. Wang, Pyridinic-N-dominated doped defective graphene as a superior oxygen electrocatalyst for ultrahigh-energy-density Zn–air batteries. *ACS Energy Lett.* **3**, 1183–1191 (2018).

20. Y. Qian, Z. Hu, X. Ge, S. Yang, Y. Peng, Z. Kang, Z. Liu, J. Y. Lee, D. Zhao, A metal-free ORR/OER bifunctional electrocatalyst derived from metal–organic frameworks for rechargeable Zn–air batteries. *Carbon* **111**, 641–650 (2017).

21. H. Li, Y. Wen, M. Jiang, Y. Yao, H. Zhou, Z. Huang, J. Li, S. Jiao, Y. Kuang, S. Luo, Understanding of neighboring Fe-N_4-C and Co-N_4-C dual active centers for oxygen reduction reaction. *Adv. Funct. Mater.* **31**, 2011289 (2021).

22. H. Liu, J. Guan, S. Yang, Y. Yu, R. Shao, Z. Zhang, M. Dou, F. Wang, Q. Xu, Metal–organic framework-derived Co_2P nanoparticle/multi-doped porous carbon as a trifunctional electrocatalyst. *Adv. Mater.* **32**, 2003649 (2020).

23. Q. Wang, L. Shang, R. Shi, X. Zhang, Y. Zhao, G. I. N. Waterhouse, L.-Z. Wu, C.-H. Tung, T. Zhang, NiFe layered double hydroxide nanoparticles on Co,N-codoped carbon nanoframes as efficient bifunctional catalysts for rechargeable zinc–air batteries. *Adv. Energy Mater.* **7**, 1700467 (2017).

24. P. Peng, L. Shi, F. Huo, C. Mi, X. Wu, S. Zhang, Z. Xiang, A pyrolysis-free path toward superiorly catalytic nitrogen-coordinated single atom. *Sci. Adv.* **5**, eaaw2322 (2019).

25. H. Shang, W. Sun, R. Sui, J. Pei, L. Zheng, J. Dong, Z. Jiang, D. Zhou, Z. Zhuang, W. Chen, J. Zhang, D. Wang, Y. Li, Engineering isolated Mn–N_2C_2 atomic interface sites for efficient bifunctional oxygen reduction and evolution reaction. *Nano Lett.* **20**, 5443–5450 (2020).

26. E. F. Holby, G. Wang, P. Zelenay, Acid stability and demetalation of PGM-free ORR electrocatalyst structures from density functional theory: A model for "single-atom catalyst" dissolution. *ACS Catal.* **10**, 14527–14539 (2020).

27. Y. Wang, D. Wang, Y. Li, Rational design of single-atom site electrocatalysts: From theoretical understandings to practical applications. *Adv. Mater.* **33**, 2008151 (2021).

28. H. Zhang, Y. Liu, T. Chen, J. Zhang, J. Zhang, X. W. Lou, Unveiling the activity origin of electrocatalytic oxygen evolution over isolated Ni atoms supported on a N-doped carbon matrix. *Adv. Mater.* **31**, 1904548 (2019).

29. Z. Li, Y. Chen, S. Ji, Y. Tang, W. Chen, A. Li, J. Zhao, Y. Xiong, Y. Wu, Y. Gong, T. Yao, W. Liu, L. Zheng, J. Dong, Y. Wang, Z. Zhuang, W. Xing, C.-T. He, C. Peng, W.-C. Cheong, Q. Li, M. Zhang, Z. Chen, N. Fu, X. Gao, W. Zhu, J. Wan, J. Zhang, L. Gu, S. Wei, P. Hu, J. Luo, J. Li, C. Chen, Q. Peng, X. Duan, Y. Huang, X.-M. Chen, D. Wang, Y. Li, Iridium single-atom catalyst on nitrogen-doped carbon for formic acid oxidation synthesized using a general host–guest strategy. *Nat. Chem.* **12**, 764–772 (2020).

30. X. Li, L. Liu, X. Ren, J. Gao, Y. Huang, B. Liu, Microenvironment modulation of single-atom catalysts and their roles in electrochemical energy conversion. *Sci. Adv.* **6**, eabb6833 (2020).

31. H. Xu, D. Cheng, D. Cao, X. C. Zeng, A universal principle for a rational design of single-atom electrocatalysts. *Nat. Catal.* **1**, 339–348 (2018).

32. Y. P. Zhu, C. Guo, Y. Zheng, S.-Z. Qiao, Surface and interface engineering of noble-metal-free electrocatalysts for efficient energy conversion processes. *Acc. Chem. Res.* **50**, 915–923 (2017).

33. W. Guo, Z. Wang, X. Wang, Y. Wu, General design concept for single-atom catalysts toward heterogeneous catalysis. *Adv. Mater.* **33**, 2004287 (2021).

34. J. Xi, H. S. Jung, Y. Xu, F. Xiao, J. W. Bae, S. Wang, Synthesis strategies, catalytic applications, and performance regulation of single-atom catalysts. *Adv. Funct. Mater.* **31**, 2008318 (2021).

35. H. Zhang, W. Zhou, T. Chen, B. Y. Guan, Z. Li, X. W. Lou, A modular strategy for decorating isolated cobalt atoms into multichannel carbon matrix for electrocatalytic oxygen reduction. *Energ. Environ. Sci.* **11**, 1980–1984 (2018).

36. M. Kuang, Q. Wang, P. Han, G. Zheng, Cu, Co-embedded N-enriched mesoporous carbon for efficient oxygen reduction and hydrogen evolution reactions. *Adv. Energy Mater.* **7**, 1700193 (2017).

37. J. Li, M. Chen, D. A. Cullen, S. Hwang, M. Wang, B. Li, K. Liu, S. Karakalos, M. Lucero, H. Zhang, C. Lei, H. Xu, G. E. Sterbinsky, Z. Feng, D. Su, K. L. More, G. Wang, Z. Wang, G. Wu, Atomically dispersed manganese catalysts for oxygen reduction in proton-exchange membrane fuel cells. *Nat. Catal.* **1**, 935–945 (2018).

38. B.-Q. Li, C.-X. Zhao, S. Chen, J.-N. Liu, X. Chen, L. Song, Q. Zhang, Framework-porphyrin-derived single-atom bifunctional oxygen electrocatalysts and their applications in Zn–air batteries. *Adv. Mater.* **31**, 1900592 (2019).

39. Y. Chen, S. Ji, Y. Wang, J. Dong, W. Chen, Z. Li, R. Shen, L. Zheng, Z. Zhuang, D. Wang, Y. Li, Isolated single iron atoms anchored on N-doped porous carbon as an efficient electrocatalyst for the oxygen reduction reaction. *Angew. Chem. Int. Ed.* **56**, 6937–6941 (2017).

40. Y. Xiong, J. Dong, Z.-Q. Huang, P. Xin, W. Chen, Y. Wang, Z. Li, Z. Jin, W. Xing, Z. Zhuang, J. Ye, X. Wei, R. Cao, L. Gu, S. Sun, L. Zhuang, X. Chen, H. Yang, C. Chen, Q. Peng, C.-R. Chang, D. Wang, Y. Li, Single-atom Rh/N-doped carbon electrocatalyst for formic acid oxidation. *Nat. Nanotechnol.* **15**, 390–397 (2020).

41. J. Xie, B.-Q. Li, H.-J. Peng, Y.-W. Song, J.-X. Li, Z.-W. Zhang, Q. Zhang, From supramolecular species to self-templated porous carbon and metal-doped carbon for oxygen reduction reaction catalysts. *Angew. Chem. Int. Ed.* **58**, 4963–4967 (2019).

42. F. Wu, C. Pan, C.-T. He, Y. Han, W. Ma, H. Wei, W. Ji, W. Chen, J. Mao, P. Yu, D. Wang, L. Mao, Y. Li, Single-atom Co–N_4 electrocatalyst enabling four-electron oxygen reduction with enhanced hydrogen peroxide tolerance for selective sensing. *J. Am. Chem. Soc.* **142**, 16861–16867 (2020).

43. P. Yin, T. Yao, Y. Wu, L. Zheng, Y. Lin, W. Liu, H. Ju, J. Zhu, X. Hong, Z. Deng, G. Zhou, S. Wei, Y. Li, Single cobalt atoms with precise N-coordination as superior oxygen reduction reaction catalysts. *Angew. Chem. Int. Ed.* **55**, 10800–10805 (2016).

44. H. C. Kolb, M. G. Finn, K. B. Sharpless, Click chemistry: Diverse chemical function from a few good reactions. *Angew. Chem. Int. Ed.* **40**, 2004–2021 (2001).

45. A. Tortajada, M. Borjesson, R. Martin, Nickel-catalyzed reductive carboxylation and amidation reactions. *Acc. Chem. Res.* **54**, 3941–3952 (2021).

46. A. Minatti, S. L. Buchwald, Synthesis of indolines via a domino Cu-catalyzed amidation/cyclization reaction. *Org. Lett.* **10**, 2721–2724 (2008).

47. V. Chandra, K. S. Kim, Highly selective adsorption of Hg^{2+} by a polypyrrole-reduced graphene oxide composite. *Chem. Commun.* **47**, 3942–3944 (2011).

48. Y. Qu, L. Wang, Z. Li, P. Li, Q. Zhang, Y. Lin, F. Zhou, H. Wang, Z. Yang, Y. Hu, M. Zhu, X. Zhao, X. Han, C. Wang, Q. Xu, L. Gu, J. Luo, L. Zheng, Y. Wu, Ambient synthesis of single-atom catalysts from bulk metal via trapping of atoms by surface dangling bonds. *Adv. Mater.* **31**, 1904496 (2019).

49. J. Li, M. T. Sougrati, A. Zitolo, J. M. Ablett, I. C. Oguz, T. Mineva, I. Matanovic, P. Atanassov, Y. Huang, I. Zenyuk, A. Di Cicco, K. Kumar, L. Dubau, F. Maillard, G. Drazic, F. Jaouen, Identification of durable and non-durable fenx sites in Fe–N–C materials for proton exchange membrane fuel cells. *Nat. Catal.* **4**, 10–19 (2021).

50. F. Morales, F. M. F. de Groot, P. Glatzel, E. Kleimenov, H. Bluhm, M. Havecker, A. Knop-Gericke, B. M. Weckhuysen, In situ x-ray absorption of Co/Mn/TiO_2 catalysts for Fischer–Tropsch synthesis. *J. Phys. Chem. B* **108**, 16201–16207 (2004).

51. W. Liu, L. Zhang, W. Yan, X. Liu, X. Yang, S. Miao, W. Wang, A. Wang, T. Zhang, Single-atom dispersed Co–N–C catalyst: Structure identification and performance for hydrogenative coupling of nitroarenes. *Chem. Sci.* **7**, 5758–5764 (2016).

52. T. Tang, W.-J. Jiang, X.-Z. Liu, J. Deng, S. Niu, B. Wang, S.-F. Jin, Q. Zhang, L. Gu, J.-S. Hu, L.-J. Wan, Metastable rock salt oxide-mediated synthesis of high-density dual-protected M@NC for long-life rechargeable zinc–air batteries with record power density. *J. Am. Chem. Soc.* **142**, 7116–7127 (2020).

53. N.-I. Kim, Y. J. Sa, T. S. Yoo, S. R. Choi, R. A. Afzal, T. Choi, Y.-S. Seo, K.-S. Lee, J. Y. Hwang, W. S. Choi, S. H. Joo, J.-Y. Park, Oxygen-deficient triple perovskites as highly active and durable bifunctional electrocatalysts for oxygen electrode reactions. *Sci. Adv.* **4**, eaap9360 (2018).

54. A. Arul, H. Pak, K. U. Moon, M. Christy, M. Y. Oh, K. S. Nahm, Metallomacrocyclic carbon complex: A study of bifunctional electrocatalytic activity for oxygen reduction and oxygen evolution reactions and their lithium–oxygen battery applications. *Appl. Catal. Environ.* **220**, 488–496 (2018).

55. S. Cai, Z. Meng, H. Tang, Y. Wang, P. Tsiakaras, 3D Co-N-doped hollow carbon spheres as excellent bifunctional electrocatalysts for oxygen reduction reaction and oxygen evolution reaction. *Appl. Catal. Environ.* **217**, 477–484 (2017).

56. S. Dilpazir, H. He, Z. Li, M. Wang, P. Lu, R. Liu, Z. Xie, D. Gao, G. Zhang, Cobalt single atoms immobilized N-doped carbon nanotubes for enhanced bifunctional catalysis toward oxygen reduction and oxygen evolution reactions. *ACS Appl. Energy Mater.* **1**, 3283–3291 (2018).

57. L. Liu, H. Su, F. Tang, X. Zhao, Q. Liu, Confined organometallic Au_1N_x single-site as an efficient bifunctional oxygen electrocatalyst. *Nano Energy* **46**, 110–116 (2018).

58. J. Sanetuntikul, S. Hyun, P. Ganesan, S. Shanmugam, Cobalt and nitrogen co-doped hierarchically porous carbon nanostructure: A bifunctional electrocatalyst for oxygen reduction and evolution reactions. *J. Mater. Chem. A* **6**, 24078–24085 (2018).

59. X. Zhang, J. Luo, H.-F. Lin, P. Tang, J. R. Morante, J. Arbiol, K. Wan, B.-W. Mao, L.-M. Liu, J. Fransaer, Tailor-made metal–nitrogen–carbon bifunctional electrocatalysts for rechargeable Zn–air batteries via controllable MOF units. *Energy Storage Mater.* **17**, 46–61 (2019).

60. J. Chen, H. Li, C. Fan, Q. Meng, Y. Tang, X. Qiu, G. Fu, T. Ma, Dual single-atomic Ni–N_4 and Fe–N_4 sitesFe□N4Sites constructing Janus hollow graphene for selective oxygen electrocatalysis. *Adv. Mater.* **32**, 2003134 (2020).

61. L. Ma, S. Chen, Z. Pei, Y. Huang, G. Liang, F. Mo, Q. Yang, J. Su, Y. Gao, J. A. Zapien, C. Zhi, Single-site active iron-based bifunctional oxygen catalyst for a compressible and rechargeable zinc–air battery. *ACS Nano* **12**, 1949–1958 (2018).

62. M. Ma, A. Kumar, D. Wang, Y. Wang, Y. Jia, Y. Zhang, G. Zhang, Z. Yan, X. Sun, Boosting the bifunctional oxygen electrocatalytic performance of atomically dispersed Fe site via atomic Ni neighboring. *Appl. Catal. Environ.* **274**, 119091 (2020).

63. S. Cai, R. Wang, W. M. Yourey, J. Li, H. Zhang, H. Tang, An efficient bifunctional electrocatalyst derived from layer-by-layer self-assembly of a three-dimensional porous Co–N–C@graphene. *Sci. Bull.* **64**, 968–975 (2019).

64. C. Du, Y. Gao, J. Wang, W. Chen, A new strategy for engineering a hierarchical porous carbon-anchored Fe single-atom electrocatalyst and the insights into its bifunctional catalysis for flexible rechargeable Zn–air batteries. *J. Mater. Chem. A* **8**, 9981–9990 (2020).

65. K. Kim, T. Kang, M. Kim, J. Kim, Three-dimensional entangled and twisted structures of nitrogen doped poly-(1,4-diethynylbenzene) chain combined with cobalt single atom as a highly efficient bifunctional electrocatalyst. *Appl. Catal. Environ.* **275**, 119107 (2020).

66. C. Tang, B. Wang, H.-F. Wang, Q. Zhang, Defect engineering toward atomic Co–N_x–C in hierarchical graphene for rechargeable flexible solid Zn–air batteries. *Adv. Mater.* **29**, 1703185 (2017).

67. L. Wei, L. Qiu, Y. Liu, J. Zhang, D. Yuan, L. Wang, Mn-doped Co–N–C dodecahedron as a bifunctional electrocatalyst for highly efficient Zn–air batteries. *ACS Sustainable Chem. Eng.* **7**, 14180–14188 (2019).

68. J. Han, X. Meng, L. Lu, J. Bian, Z. Li, C. Sun, Single-atom Fe–N_x–C as an efficient electrocatalyst for zinc–air batteries. *Adv. Funct. Mater.* **29**, 1808872 (2019).

69. Y. Guo, P. Yuan, J. Zhang, Y. Hu, I. S. Amiinu, X. Wang, J. Zhou, H. Xia, Z. Song, Q. Xu, S. Mu, Carbon nanosheets containing discrete Co–N_x–B_y–C active sites for efficient oxygen electrocatalysis and rechargeable Zn–air batteries. *ACS Nano* **12**, 1894–1901 (2018).

70. B.-Q. Li, S.-Y. Zhang, X. Chen, C.-Y. Chen, Z.-J. Xia, Q. Zhang, One-pot synthesis of framework porphyrin materials and their applications in bifunctional oxygen electrocatalysis. *Adv. Funct. Mater.* **29**, 1901301 (2019).

71. J. Zhang, M. Zhang, Y. Zeng, J. Chen, L. Qiu, H. Zhou, C. Sun, Y. Yu, C. Zhu, Z. Zhu, Single Fe atom on hierarchically porous S, N-codoped nanocarbon derived from porphyra enable boosted oxygen catalysis for rechargeable Zn–air batteries. *Small* **15**, 1900307 (2019).

72. H. Qin, Y. Wang, B. Wang, X. Duan, H. Lei, X. Zhang, H. Zheng, W. Zhang, R. Cao, Cobalt porphyrins supported on carbon nanotubes as model catalysts of metal–N_4/C sites for oxygen electrocatalysis. *J. Energy Chem.* **53**, 77–81 (2021).

73. L. Xie, X.-P. Zhang, B. Zhao, P. Li, J. Qi, X. Guo, B. Wang, H. Lei, W. Zhang, U.-P. Apfel, R. Cao, Enzyme-inspired iron porphyrins for improved electrocatalytic oxygen reduction and evolution reactions. *Angew. Chem. Int. Ed.* **60**, 7576–7581 (2021).

74. Y. Bu, O. Gwon, G. Nam, H. Jang, S. Kim, Q. Zhong, J. Cho, G. Kim, A highly efficient and robust cation ordered perovskite oxide as a bifunctional catalyst for rechargeable zinc–air batteries. *ACS Nano* **11**, 11594–11601 (2017).

75. F. W. T. Goh, Z. Liu, X. Ge, Y. Zong, G. Du, T. S. A. Hor, Ag nanoparticle-modified MnO_2 nanorods catalyst for use as an air electrode in zinc–air battery. *Electrochim. Acta* **114**, 598–604 (2013).

76. G. Du, X. Liu, Y. Zong, T. S. A. Hor, A. Yu, Z. Liu, Co_3O_4 nanoparticle-modified MnO_2 nanotube bifunctional oxygen cathode catalysts for rechargeable zinc–air batteries. *Nanoscale* **5**, 4657–4661 (2013).

77. L. An, B. Huang, Y. Zhang, R. Wang, N. Zhang, T. Dai, P. Xi, C.-H. Yan, Interfacial defect engineering for improved portable zinc–air batteries with a broad working temperature. *Angew. Chem. Int. Ed.* **58**, 9459–9463 (2019).

78. Y. Zhan, G. Du, S. Yang, C. Xu, M. Lu, Z. Liu, J. Y. Lee, Development of cobalt hydroxide as a bifunctional catalyst for oxygen electrocatalysis in alkaline solution. *ACS Appl. Mater. Interfaces* **7**, 12930–12936 (2015).

79. J. Wang, H. Wu, D. Gao, S. Miao, G. Wang, X. Bao, High-density iron nanoparticles encapsulated within nitrogen-doped carbon nanoshell as efficient oxygen electrocatalyst for zinc–air battery. *Nano Energy* **13**, 387–396 (2015).

80. J. Ran, T. Wang, J. Zhang, Y. Liu, C. Xu, S. Xi, D. Gao, Modulation of electronics of oxide perovskites by sulfur doping for electrocatalysis in rechargeable Zn–air batteries. *Chem. Mater.* **32**, 3439–3446 (2020).

81. S. Peng, X. Han, L. Li, S. Chou, D. Ji, H. Huang, Y. Du, J. Liu, S. Ramakrishna, Electronic and defective engineering of electrospun $CaMnO_3$ nanotubes for enhanced oxygen electrocatalysis in rechargeable zinc–air batteries. *Adv. Energy Mater.* **8**, 1800612 (2018).

82. W. Li, Y. Li, H. Fu, G. Yang, Q. Zhang, S. Chen, F. Peng, Phosphorus doped Co_9S_8@CS as an excellent air-electrode catalyst for zinc–air batteries. *Chem. Eng. J.* **381**, 122683 (2020).

83. S. Ibraheem, S. Chen, J. Li, Q. Wang, Z. Wei, In situ growth of vertically aligned FeCoOOH-nanosheets/nanoflowers on Fe,N co-doped 3D-porous carbon as efficient bifunctional electrocatalysts for rechargeable zinc–O_2 batteries. *J. Mater. Chem. A* **7**, 9497–9502 (2019).

84. G. Fu, Z. Cui, Y. Chen, Y. Li, Y. Tang, J. B. Goodenough, Ni_3Fe-N doped carbon sheets as a bifunctional electrocatalyst for air cathodes. *Adv. Energy Mater.* **7**, 1601172 (2017).

85. D. Ji, J. Sun, L. Tian, A. Chinnappan, T. Zhang, W. A. D. M. Jayathilaka, R. Gosh, C. Baskar, Q. Zhang, S. Ramakrishna, Engineering of the heterointerface of porous carbon nanofiber-supported nickel and manganese oxide nanoparticle for highly efficient bifunctional oxygen catalysis. *Adv. Funct. Mater.* **30**, 1910568 (2020).

86. T. Sun, J. Wang, C. Qiu, X. Ling, B. Tian, W. Chen, C. Su, B, N codoped and defect-rich nanocarbon material as a metal-free bifunctional electrocatalyst for oxygen reduction and evolution reactions. *Adv. Sci.* **5**, 1800036 (2018).

87. K. Wan, J. Luo, X. Zhang, C. Zhou, J. W. Seo, P. Subramanian, J.-W. Yan, J. Fransaer, A template-directed bifunctional NiS_x/nitrogen-doped mesoporous carbon electrocatalyst for rechargeable Zn–air batteries. *J. Mater. Chem. A* **7**, 19889–19897 (2019).

88. Y. Tang, F. Jing, Z. Xu, F. Zhang, Y. Mai, D. Wu, Highly crumpled hybrids of nitrogen/sulfur dual-doped graphene and Co_9S_8 nanoplates as efficient bifunctional oxygen electrocatalysts. *ACS Appl. Mater. Interfaces* **9**, 12340–12347 (2017).

89. Z. Rong, C. Dong, S. Zhang, W. Dong, F. Huang, $Co_{5.47}N$ loaded N-doped carbon as an efficient bifunctional oxygen electrocatalyst for a Zn–air battery. *Nanoscale* **12**, 6089–6095 (2020).

90. X. Wang, Y. Li, T. Jin, J. Meng, L. Jiao, M. Zhu, J. Chen, Electrospun thin-walled $CuCo_2O_4$@C nanotubes as bifunctional oxygen electrocatalysts for rechargeable Zn–air batteries. *Nano Lett.* **17**, 7989–7994 (2017).

91. L. Wei, H. E. Karahan, S. Zhai, H. Liu, X. Chen, Z. Zhou, Y. Lei, Z. Liu, Y. Chen, Amorphous bimetallic oxide-graphene hybrids as bifunctional oxygen electrocatalysts for rechargeable Zn–air batteries. *Adv. Mater.* **29**, 1701410 (2017).

92. H.-F. Wang, C. Tang, B. Wang, B.-Q. Li, X. Cui, Q. Zhang, Defect-rich carbon fiber electrocatalysts with porous graphene skin for flexible solid-state zinc–air batteries. *Energy Storage Mater.* **15**, 124–130 (2018).
